# Functional and genetic divergence of aging-related *TOMM40* polymorphisms in Alzheimer’s disease: an integrative bioinformatics and systematic review with meta-analysis and trial sequential analysis

**DOI:** 10.3389/fnins.2026.1772368

**Published:** 2026-04-10

**Authors:** Shiqi Li, Yang Luo, Shasha Shi, Yan Ding

**Affiliations:** 1 Department of Clinical Laboratory, Ningbo Psychiatric Hospital (The Affiliated Minkang Hospital of Ningbo University), Ningbo, China; 2 Department of Cardiovascular Medicine, Province Wuyi County First People's Hospital, Zhejiang, China; 3 Department of Clinical Laboratory, Province Wuyi County First People's Hospital, Zhejiang, China; 4 Department of Clinical Laboratory, Chongqing Emergency Medical Center, School of Medicine, Chongqing University Central Hospital, Chongqing University, Chongqing, China

**Keywords:** aging, Alzheimer’s disease, meta-analysis, mitochondrial dysfunction, polymorphism, TOMM40

## Abstract

**Background:**

The etiology of late-onset Alzheimer’s disease (AD) is only partly understood. Because *TOMM40* is located within the *APOE–TOMM40–APOC1* locus, its independent role remains unclear. This study aimed to assess the association of six *TOMM40* polymorphisms with AD risk across five genetic models while integrating genome-wide, regulatory, and functional genomic evidence to clarify their potential biological roles.

**Methods:**

A comprehensive literature search was conducted across five electronic databases. RevMan 5.1 was used for meta-analysis, including subgroup, meta-regression, and sensitivity analyses. To provide biological context, genome-wide data from IGAP/NIAGADS, AD-specific functional annotations from AGORA, and regulatory eQTL/sQTL evidence from GTEx were incorporated, with pathway enrichment using Enrichr.

**Results:**

Thirteen articles were included in the meta-analysis. *rs2075650* showed a significantly increased AD risk across all genetic models, while *rs157580* consistently demonstrated a protective effect. *rs157581* was also associated with elevated risk, whereas *rs8106922, rs11556505, and rs1160985* showed no significant associations. Bioinformatic analysis showed that *rs2075650* and *rs157581* reside within the APOE-linked LD block and affect *TOMM40* splicing, whereas rs157580 demonstrated an LD-independent regulatory pattern, influencing the expression of genes involved in lipid- and amyloid-related pathways.

**Conclusion:**

*rs2075650*, *rs157580*, and *rs157581* show significant associations with AD risk. *rs2075650* and rs157581 confer elevated risk, while *rs157580* is protective. Integrated genomic evidence indicates that the risk variants act via *TOMM40* splicing within the *APOE* locus, whereas the protective variant modulates expression of lipid- and amyloid-related genes, suggesting distinct mechanisms.

## 1 Introduction

Alzheimer’s disease (AD) is the most prevalent type of dementia, accounting for at least two-thirds of cases in individuals aged 65 and older (Kumar et al., 2025; Breijyeh and Karaman, 2020). AD is a neurodegenerative condition with insidious onset and progressive impairment of behavioral and cognitive functions (Breijyeh and Karaman, 2020; Annicchiarico et al., 2007). These functions include memory, comprehension, language, attention, reasoning, and judgment (Kumar et al., 2025; Atri et al., 2024). While AD does not directly cause death, it substantially raises vulnerability to other complications, which can eventually lead to a person’s death (Kumar et al., 2025). Between 2000 and 2019, reported deaths from AD increased by more than 145% (Better, 2023).

Significant progress has been made in developing biomarkers for specific and early diagnosis of AD over the past decade (Blennow and Zetterberg, 2018). These biomarkers include neuroimaging markers obtained through amyloid and tau PET scans, cerebrospinal fluid (CSF), and plasma markers, such as amyloid, tau, and phospho-tau levels (Kumar et al., 2025; Prvulovic and Hampel, 2011; Meyer et al., 2020).

Linkage studies, candidate gene, and whole-genome association studies have resulted in a tremendous amount of putative risk genes for AD (Bettens et al., 2010). AD is a genetically complex disorder associated with multiple genetic defects, either mutational or of susceptibility (Cacabelos et al., 2005). The application of functional genomics to AD can be a suitable strategy for molecular diagnosis and for understanding pathophysiological mechanisms associated with AD-related neurodegeneration (Cacabelos et al., 2005). The search for biomarkers for cognitive aging has been active, and the same biomarkers for AD are also commonly employed in cognitive aging research due to a high prevalence of AD in older adults (Jack Jr et al., 2016). While several encouraging findings on the relationship between the AD-associated genes and cognitive aging have emerged, human data are scarce in terms of single-nucleotide polymorphisms (SNPs) (Lin et al., 2017).

The *TOMM40* gene maps on chromosome 19q13.32 and encodes the TOM40 protein, which is a subunit of the Translocase of Mitochondrial Outer Membrane (TOM) complex (Humphries et al., 2005). Several genetic association studies have revealed a strong link between genetic variants in multiple loci and exceptional human longevity (Liu et al., 2021), and *TOMM40* was identified as one of the candidate genes.

Two meta-analyses reported the association of *TOMM40* polymorphisms of *rs157580* and *rs2075650* (Bao et al., 2016) and *rs2075650* alone (He et al., 2016) with the risk of AD without reporting any genetic model.

We aimed to evaluate the association of six *TOMM40* polymorphisms (*rs2075650*, *rs157580*, *rs157581*, *rs8106922*, *rs11556505*, and *rs1160985*) with susceptibility to AD across five genetic models, and to complement these findings with genome-wide and functional genomic analyses to clarify their potential biological relevance.

## 2 Materials and methods

### 2.1 Bioinformatic analysis

To complement the meta-analysis and provide genomic and functional context for *TOMM40* polymorphisms, we incorporated several publicly available datasets.

#### 2.1.1 Genome-wide and locus-level genetic data

Genome-wide association statistics were obtained from the IGAP Stage 1 dataset through the NIAGADS database (https://www.niagads.org). For each SNP located within the *TOMM40* gene, we extracted the genomic location, variant consequence, linkage disequilibrium (LD) structure, and GWAS association *p*-values from the IGAP Stage 1 dataset in NIAGADS. To visualize the exact genomic positions of these *TOMM40* SNPs, we used the UCSC Genome Browser (https://genome.ucsc.edu). For genomic annotation, the term “gene body” refers to the complete transcribed region of *TOMM40*, including all exons, introns, and untranslated regions (5′ and 3′ UTRs), bounded by the transcription start site and transcription termination site according to the GRCh38 genome assembly.

#### 2.1.2 Alzheimer’s disease–specific biological domain classification and gene ontology mapping

*TOMM40* biological domain classification was obtained from the AD Knowledge Portal—AGORA (https://agora.adknowledgeportal.org). The Biological Domain framework is an AD–specific system developed by the TREAT-AD Center, which assigns genes to 19 AD-related biological domains. Although the domains themselves are AD-specific, the assignment of genes to domains is performed using curated Gene Ontology (GO) term mappings. These curated GO annotations link *TOMM40* to its corresponding AD-related biological pathways.

#### 2.1.3 eQTL and sQTL analyses

To explore potential mechanisms underlying *TOMM40* polymorphisms, eQTL and sQTL data for SNPs identified as significant in the meta-analysis were obtained from the GTEx v8 Portal (https://gtexportal.org). For each SNP, brain-derived tissues were prioritized to reflect AD–relevant biology. If no brain-specific QTL was available, the tissue with the largest absolute normalized effect size (NES) across other tissues was selected. Associations were considered significant at *p* < 0.05, and genes or splice junctions with more than three significant associations were submitted to pathway enrichment analyses using Enrichr (https://maayanlab.cloud/Enrichr/) (Elsevier Pathways, Reactome, Gene Ontology Biological Processes). This approach ensures focus on biologically relevant tissues and robust SNP–gene associations.

### 2.2 Systematic review and meta-analysis

#### 2.2.1 Study design

The meta-analysis was managed following the protocols of the Preferred Reporting Items for Systematic Reviews and Meta-Analyses (PRISMA) (Page et al., 2021). PECO framework included: Population: Individuals, particularly elderly adults, at risk of developing AD. Exposure: Single-nucleotide polymorphisms of the *TOMM40* gene that are related to aging. Comparator: Individuals with and without AD. Outcome: Prevalence of alleles and genotypes of the *TOMM40* gene. The study has not been registered in any databases.

#### 2.2.2 Literature search

Five electronic databases—PubMed, Web of Science, Scopus, Cochrane Library, and CNKI (China National Knowledge Infrastructure)—were searched up to November 6, 2025, without any restrictions. The search terms used were (“TOMM40” or “translocase of outer mitochondrial membrane 40”) and (“Alzheimer disease” or “Alzheimer’s disease”) and (“polymorphism” or “genotype” or “variant” or “allele”). We also examined references from eligible studies and manually reviewed articles to identify relevant publications, supplementing our search with electronic sources like Google Scholar. Notably, ethical approval was not required for this study, as it only involved retrieving and synthesizing data from previously published articles.

#### 2.2.3 Eligibility criteria

Eligible studies included case–control, cross-sectional, or cohort investigations reporting genotype or allele frequency data for *TOMM40* polymorphisms (*rs2075650, rs157580, rs157581, rs8106922, rs11556505,* and *rs1160985*) in patients with AD and cognitively healthy control subjects.

Studies were required to meet the following inclusion criteria:

1. Participants diagnosed with AD using established clinical or neuropathological diagnostic criteria (e.g., NINCDS-ADRDA or comparable internationally accepted standards);
2. Control subjects were required to be cognitively normal individuals without a clinical diagnosis of AD or other neurodegenerative disorders.
3. Availability of sufficient genotype or allele frequency data to calculate ORs and 95% CIs. Because the meta-analysis applied homozygous, heterozygous, dominant, recessive, and allelic genetic models, only biallelic polymorphisms with three possible genotypes (e.g., AA, AB, BB) and extractable genotype frequency data were eligible for inclusion. Multi-allelic polymorphisms (such as variable-length polymorphisms) were therefore excluded.
4. Genotyping performed using validated molecular methods (e.g., PCR-based assays, TaqMan assays, sequencing, or array-based platforms);
5. Independent study populations without overlapping datasets.

Studies were excluded if:

1. Participants had major neurological or systemic disorders that could confound AD diagnosis;
2. Genotype data were incomplete or unavailable;
3. They were genome-wide association studies lacking extractable genotype frequency data;
4. They were reviews, editorials, conference abstracts, or previous meta-analyses.

#### 2.2.4 Data extraction

To ensure consistency in the screening criteria and data collection process, two authors independently conducted a review of the literature and data extraction.

#### 2.2.5 Quality assessment of methodological rigor

The methodological rigor and risk of bias of the included observational studies were independently evaluated by two reviewers using the Newcastle–Ottawa Scale (NOS) for case–control studies. The NOS assesses study quality across three domains: (Kumar et al., 2025) selection of cases and controls (maximum 4 stars), (Breijyeh and Karaman, 2020) comparability between study groups (maximum 2 stars), and (Annicchiarico et al., 2007) exposure assessment (maximum 3 stars), with a maximum score of 9 stars. Studies scoring 7–9 stars were considered high quality, 5–6 moderate quality, and ≤4 low quality. Disagreements between reviewers were resolved through discussion and consensus. The resulting quality scores were incorporated into meta-regression analyses to evaluate their influence on pooled effect estimates.

#### 2.2.6 Statistical analysis

Meta-analysis was performed using Review Manager Version 5.1 (RevMan 5.1) software, and pooled effect estimates were expressed as odds ratios (ORs) with 95% confidence intervals (CIs). Between-study heterogeneity was assessed using Cochran’s Q test and quantified using the I^2^ statistic. A random-effects model was applied when significant heterogeneity was detected (P*_heterogeneity_* < 0.10 or I^2^ > 50%) to account for between-study variability; otherwise, a fixed-effect model was used (Sadafi et al., 2024; Imani et al., 2022).

To investigate potential sources of heterogeneity and effect modification, predefined subgroup analyses were conducted when sufficient studies were available (≥3 studies per subgroup). Subgroups were stratified according to ethnicity (e.g., Caucasian and Asian populations) and study sample size. In addition, meta-regression analyses were performed using Comprehensive Meta-Analysis Version 2.0 (CMA 2.0) software to evaluate the influence of publication year, sample size, and methodological quality score on pooled effect estimates.

Sensitivity analyses, including one-study-removed and cumulative analyses, were conducted to assess the robustness and stability of pooled outcomes and to identify potential influential studies contributing to heterogeneity.

Publication bias was evaluated using funnel plot symmetry together with Begg’s and Egger’s regression tests, with a significance threshold of *p* < 0.10 (Golshah et al., 2024; Mohammadi et al., 2023). Additionally, subgroup, meta-regression, and sensitivity analyses were performed. Meta-regression and sensitivity analyses were performed by the Comprehensive Meta-Analysis Version 2.0 (CMA 2.0) software.

Trial sequential analysis (TSA) was conducted using TSA software (version 0.9.5.10 beta) (Wetterslev et al., 2017) to assess the sufficiency and reliability of cumulative evidence. The required information size (RIS) for each polymorphism was calculated assuming an alpha risk of 5% and a beta risk of 20% (Mohammadi et al., 2021; Golshah et al., 2021). Crossing of the cumulative Z-curve over the RIS boundary was interpreted as sufficient evidence supporting the reliability of pooled conclusions (Hatami et al., 2024; Imani et al., 2024).

## 3 Results

### 3.1 Genome-wide and TOMM40-proximal genetic associations with Alzheimer’s disease

**Figure 1:**
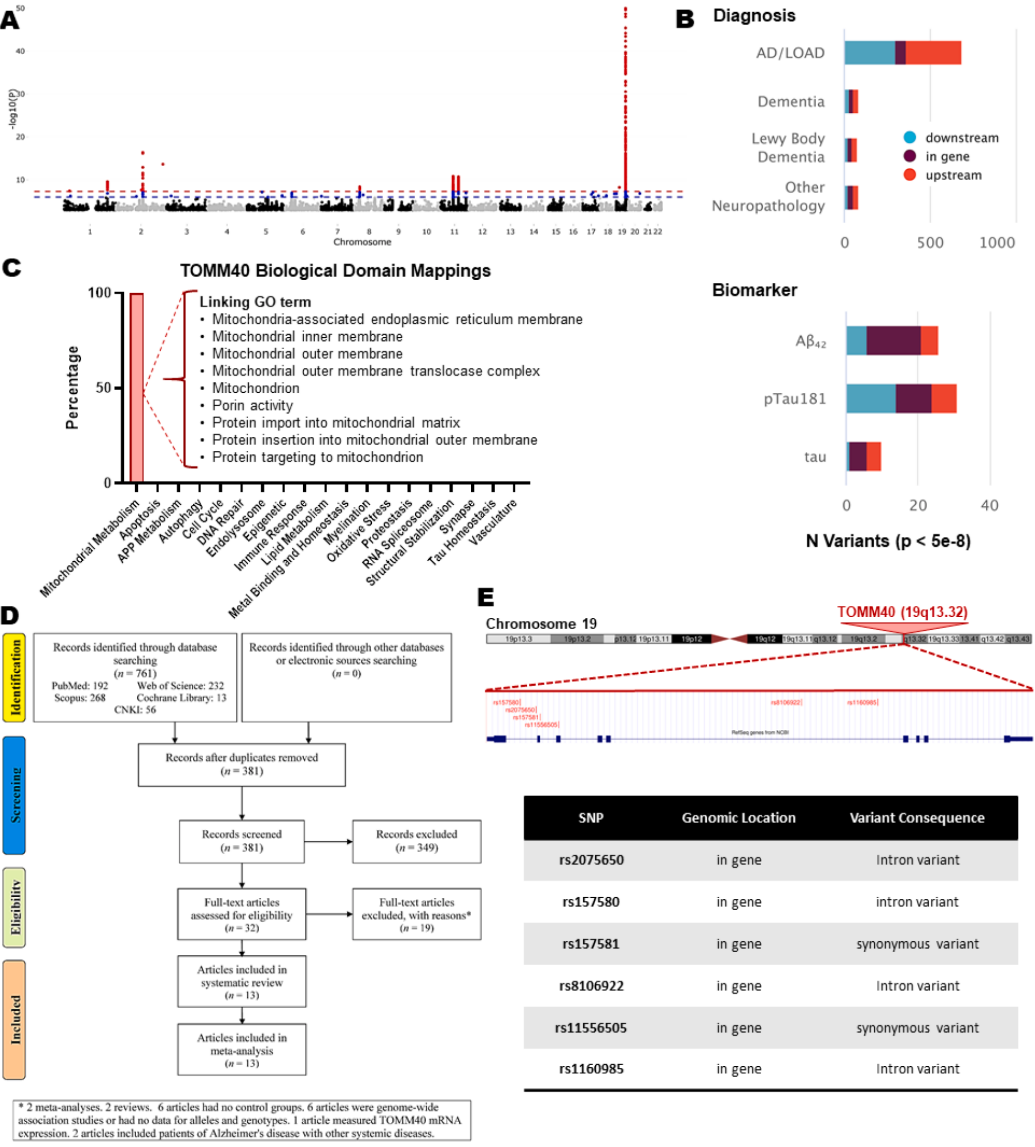
Genome-wide, locus-level, functional, and study-selection overview for *TOMM40* polymorphisms included in this analysis. **(A)** Manhattan plot of genome-wide association results from the IGAP Stage 1 dataset (NIAGADS), showing −log_10_ (*p*-values) across all chromosomes. Chromosome 19 displays a dense cluster of genome-wide significant variants corresponding to the *APOE–TOMM40–APOC1* locus. **(B)** Locus-level summary of variants located within or surrounding the *TOMM40* genomic locus derived from NIAGADS and grouped by phenotype category. Diagnosis panel: number of genome-wide significant variants (*p* < 5 × 10^−8^) associated with AD/LOAD, dementia, Lewy body dementia, and neuropathological outcomes. *TOMM40*-proximal variants were defined as SNPs located within the *TOMM40* gene body or within ±100 kb upstream or downstream of the gene boundaries based on the GRCh38 genome assembly. Variants were classified as upstream, intragenic, or downstream relative to *TOMM40* transcriptional coordinates. Biomarker panel: significant associations of variants located within this defined *TOMM40* locus (“*TOMM40* region”) with AD biomarkers, including cerebrospinal fluid Aβ_42_, pTau181, and total tau. **(C)** Biological domain classification of *TOMM40* from the AGORA knowledge portal. *TOMM40* shows 100% enrichment in mitochondrial-related biological domains, with GO terms mapping to mitochondrial membranes, the TOM translocase complex, protein import pathways, and porin activity. **(D)** PRISMA flow diagram summarizing the study selection process. Out of 761 records identified, 381 remained after duplicate removal; 32 full-text articles were assessed for eligibility, and 13 met inclusion criteria for qualitative and quantitative synthesis. **(E)** Genomic positioning of the six *TOMM40* polymorphisms included in the meta-analysis, shown on chromosome 19 using the UCSC Genome Browser. The table summarizes SNP genomic location and annotated variant consequences (intronic or synonymous variants). AD, Alzheimer’s disease; LOAD, late-onset Alzheimer’s disease; SNP, single-nucleotide polymorphism.

To obtain an overview of the genetic landscape underlying AD, we first examined genome-wide association results from the IGAP Stage 1 dataset in NIAGADS (Figure 1A). The Manhattan plot shows that although several chromosomes harbor significant variants, chromosome 19 exhibits an exceptionally strong and dense cluster of genome-wide significant signals. This region corresponds to the well-known *APOE–TOMM40–APOC1* locus, which has repeatedly emerged as the most prominent susceptibility region in AD.

To further characterize contributions specifically related to *TOMM40*, we next reviewed NIAGADS locus-level summary statistics for variants located upstream, within, and downstream of the gene (Figure 1B). In the Diagnosis panel, numerous *TOMM40*-proximal variants demonstrate strong associations with clinical AD, late-onset AD (LOAD), and other dementia-related phenotypes. In the Biomarker panel, variants across the *TOMM40* region also show significant associations with core AD biomarkers—including cerebrospinal fluid Aβ₄₂, pTau181, and total tau—highlighting the biological relevance of this locus not only to disease diagnosis but also to molecular pathology.

To contextualize these genetic associations within *TOMM40*’s biological role, we examined curated Gene Ontology annotations from the AGORA knowledge portal (Figure 1C). *TOMM40* displayed exclusive enrichment (100%) in mitochondrial metabolism pathways, with gene ontology terms mapping to mitochondrial inner and outer membranes, the mitochondrial translocase complex, protein import into the mitochondrial matrix, and porin activity. This functional profile aligns with *TOMM40*’s established role in mitochondrial protein translocation and suggests that genetic variation in this region may influence AD risk through mitochondrial dysfunction.

Together, these genome-wide, locus-specific, and functional annotations indicate that *TOMM40* is embedded within the most genetically active region associated with AD and functions as part of a broader susceptibility architecture rather than being driven by a single pathogenic variant. Motivated by this multilayered evidence, we systematically identified all *TOMM40*-related SNPs reported in the literature and.

### 3.2 Study selection

Figure 1D shows the selection process for a systematic review and meta-analysis. It starts with 761 records identified from five databases, which are then narrowed down to 381 records after removing duplicates. From these, 349 records are excluded during the screening phase. The remaining 32 full-text articles are assessed for eligibility, but 19 are excluded for reasons such as being meta-analyses, reviews, or lacking control groups. Ultimately, 13 articles (Bagnoli et al., 2013; Chen et al., 2023; Elias-Sonnenschein et al., 2013; Hajilou et al., 2024; Kan et al., 2015; Kim et al., 2017; Liang et al., 2022; Ma et al., 2013; Nurmaimaiti et al., 2020; Omoumi et al., 2014; Prendecki et al., 2018; Soyal et al., 2020; Takei et al., 2009) are included in both the systematic review and the meta-analysis.

### 3.3 Characteristics of the articles

The meta-analysis encompasses 13 studies published over a span of 16 years, from 2009 to 2024, with each study reporting at least one genetic polymorphism (Table 1). The studies originate from various countries, including Italy, Taiwan, Iran, and China, and involve different ethnic groups such as Caucasian, Asian, and Mixed populations. The number of cases and controls varies across the studies. Additionally, the *p*-values for Hardy–Weinberg equilibrium in control groups are provided, indicating whether the genotype frequencies are in equilibrium, and each study is assigned a quality score ranging from 6 to 9, reflecting their methodological rigor. The specific polymorphisms reported include *rs2075650* by 10 studies, *rs157580* by 6 studies, *rs157581* by 3 studies, *rs8106922* by 3 studies, and both *rs11556505* and *rs1160985* by 2 studies each. To contextualize the nature of these variants, Figure 1E illustrates their genomic positions within the *TOMM40* gene together with their annotated variant consequences. All six polymorphisms fall within the gene body and are classified as either intronic variants or synonymous coding variants.

**Table 1:** Characteristics of the studies entered into the meta-analysis.

First author, publication year	Country	Ethnicity	No. of cases/controls	Polymorphism (s)	*p*-value of HWE in the control group	Quality score
Bagnoli et al. (2013)	Italy	Caucasian	280/272	*rs2075650*	0.609	9
			262/269	*rs157580*	0.446	
			282/270	*rs157581*	0.257	
Chen et al. (2023)	Taiwan	Asian	393/213	*rs157581*	0.715	8
				*rs11556505*	0.569	
Elias-Sonnenschein et al. (2013)	Caucasian	Caucasian	884/689	*rs2075650*	**0.046**	6
			869/683	*rs157580*	0.994	
			986/695	*rs8106922*	0.004	
Hajilou et al. (2024)	Iran	Asian	117/131	*rs157580*	0.147	8
				*rs8106922*	**< 0.001**	
Kan et al. (2015)	China	Asian	200/1455	*rs2075650*	0.992	8
Kim et al. (2017)	Korea	Asian	11/12	*rs2075650*	0.752	7
Liang et al. (2022)	China	Asian	182/4694	*rs2075650*	0.742	7
Ma et al. (2013)	China	Asian	787/791	*rs2075650*	0.186	9
				*rs157580*	0.316	
				*rs1160985*	0.131	
				*rs11556505*	0.186	
Nurmaimaiti et al. (2020)	China	Asian	128/125	*rs2075650*	0.534	8
Omoumi et al. (2014)	Canada	Mixed	428/524	*rs2075650*	0.589	8
Prendecki et al. (2018)	Poland	Caucasian	88/142	*rs2075650*	**0.032**	7
Soyal et al. (2020)	Austria	Caucasian	144/150	*rs2075650*	0.767	7
				*rs157580*	0.178	
				*rs8106922*	0.339	
Takei et al. (2009)	Japan	Asian	539/700	*rs157580*	0.913	7
			535/691	*rs157581*	0.456	
			543/703	*rs1160985*	0.473	

Bold number means statistically significant (*p* < 0.05).

### 3.4 Pooled analysis

Table 2 summarizes the forest plots for various genetic polymorphisms analyzed in multiple studies and their association with the risk of AD. For *rs2075650*, 10 studies showed an elevated risk across all genetic models, with *p*-values indicating strong statistical significance and varying levels of heterogeneity for AD. For *rs157580*, 6 studies consistently indicated a protective effect (OR < 1), with high statistical significance and moderate heterogeneity for AD. *Rs8106922*, analyzed in 3 studies, shows no significant associations and high heterogeneity for AD. *Rs157581*, from 3 studies, demonstrated an elevated risk with high statistical significance and moderate heterogeneity for AD. *Rs11556505*, analyzed in 2 studies, shows no significant associations with high heterogeneity with AD. Lastly, *rs1160985*, also from 2 studies, showed no significant associations with high heterogeneity with AD. The forest plots located in Supplementary Figures S1–30 provide detailed visual representations of these associations.

**Table 2:** Summary of the forest plots.

Polymorphism	Genetic model	Number of studies	OR	95%CI		Z-value	*p*-value	I^2^
				Min.	Max.			
*rs2075650*	G vs. A	10	1.85	1.35	2.54	3.81	**0.0001**	88%
	GG vs. AA	10	4.96	3.62	6.80	9.96	**< 0.0001**	41%
	AG vs. AA	10	1.93	1.36	2.74	3.68	**0.0002**	86%
	GG + AG vs. AA	10	1.94	1.36	2.77	3.62	**0.0003**	88%
	GG vs. AA + AG	10	3.74	2.72	5.14	8.14	**< 0.00001**	17%
*rs157580*	G vs. A	6	0.66	0.60	0.71	10.06	**< 0.00001**	49%
	GG vs. AA	6	0.44	0.32	0.60	25.23	**< 0.00001**	52%
	AG vs. AA	6	0.68	0.60	0.77	6.06	**< 0.00001**	40%
	GG + AG vs. AA	6	0.60	0.53	0.68	8.27	**< 0.00001**	30%
	GG vs. AA + AG	6	0.54	0.47	0.63	7.87	**< 0.00001**	41%
*rs8106922*	G vs. A	3	1.35	0.23	7.93	0.33	0.74	99%
	GG vs. AA	3	1.63	0.08	34.69	0.31	0.75	98%
	AG vs. AA	3	1.24	0.23	6.72	0.25	0.81	98%
	GG + AG vs. AA	3	1.31	0.18	9.51	0.27	0.79	99%
	GG vs. AA + AG	3	1.54	0.14	16.61	0.35	0.72	97%
*rs157581*	C vs. T	3	1.93	1.56	2.39	6.01	**< 0.00001**	58%
	CC vs. TT	3	3.60	2.62	4.94	7.94	**< 0.00001**	24%
	TC vs. TT	3	1.95	1.42	2.68	4.10	**< 0.0001**	66%
	CC + TC vs. TT	3	2.17	1.60	2.93	5.00	**< 0.00001**	66%
	CC vs. TT + TC	3	2.74	2.02	3.71	6.49	**< 0.00001**	0%
*rs11556505*	T vs. C	2	3.60	0.61	21.17	1.42	0.16	97%
	TT vs. CC	2	6.04	0.10	355.01	0.86	0.39	82%
	CT vs. CC	2	4.08	0.87	19.04	1.79	0.07	96%
	CC + CT vs. CC	2	4.00	0.63	25.46	1.47	0.14	97%
	CC vs. TT + CT	2	4.51	0.12	170.95	0.81	0.42	78%
*rs1160985*	T vs. C	2	0.88	0.58	1.36	0.57	0.57	92%
	TT vs. CC	2	0.76	0.38	1.49	0.81	0.42	80%
	CT vs. CC	2	0.91	0.53	1.57	0.34	0.73	92%
	CC + CT vs. CC	2	0.88	0.50	1.54	0.44	0.66	93%
	CC vs. TT + CT	2	0.79	0.51	1.24	1.02	0.31	57%

Bold number means statistically significant (*p* < 0.05).

### 3.5 Subgroup analysis

The *rs2075650* polymorphism had sufficient studies compared to others for subgroup analysis. The subgroup analysis for the association of the *rs2075650* polymorphism and the risk of AD reveals that this variant shows significantly elevated risks across various genetic models, particularly in Caucasian populations and larger sample sizes (Table 3). In the G vs. A model, Caucasian studies demonstrate a notably elevated risk with low heterogeneity, whereas Asian studies do not show a significant association and exhibit higher heterogeneity. Similarly, for the GG vs. AA model, Caucasian studies indicate a highly elevated risk with no heterogeneity, while Asian studies lack significant findings. The AG vs. AA and GG vs. AA + AG models also display consistently elevated risks in Caucasian populations. The findings from studies with sample sizes of 500 or more consistently show significantly elevated risks across multiple genetic models, suggesting that the *rs2075650* polymorphism has sufficient data to support these associations. Overall, these results highlight the importance of the *rs2075650* polymorphism in genetic susceptibility, particularly among Caucasians and larger study samples.

**Table 3:** Subgroup analysis for the *rs2075650* polymorphism.

Genetic model	Variable (No. of studies)	OR	95%CI		*p*-value	I^2^
			Min.	Max.		
G vs. A	Ethnicity					
	Caucasian (4)	2.85	2.46	3.29	**< 0.00001**	25%
	Asian (5)	1.17	0.84	1.62	0.35	66%
	Sample Size					
	≥ 500 (6)	1.76	1.20	2.58	**0.004**	92%
	< 500 (4)	2.08	1.01	4.27	0.05	79%
GG vs. AA	Ethnicity					
	Caucasian (4)	6.44	4.24	9.77	**< 0.00001**	0%
	Asian (5)	1.39	0.40	4.83	0.60	55%
	Sample Size					
	≥ 500 (6)	3.92	2.14	7.18	**< 0.00001**	51%
	< 500 (4)	4.99	2.08	11.99	**0.0003**	42%
AG vs. AA	Ethnicity					
	Caucasian (4)	3.19	2.67	3.82	**< 0.00001**	0%
	Asian (5)	1.16	0.82	1.64	0.40	62%
	Sample Size					
	≥ 500 (6)	1.94	1.26	2.97	**0.002**	91%
	< 500 (4)	1.93	0.95	3.89	0.07	69%
GG + AG vs. AA	Ethnicity					
	Caucasian (4)	3.20	2.69	3.80	**< 0.00001**	43%
	Asian (5)	1.18	0.84	1.67	0.35	64%
	Sample Size					
	≥ 500 (6)	1.86	1.21	2.88	**0.005**	92%
	< 500 (4)	2.14	0.99	4.64	0.05	77%
GG vs. AA + AG	Ethnicity					
	Caucasian (4)	4.44	2.94	6.71	**< 0.00001**	7.10
	Asian (5)	1.39	0.41	4.71	0.60	54%
	Sample Size					
	≥ 500 (6)	3.67	2.61	5.16	**< 0.00001**	29%
	< 500 (4)	4.21	1.76	10.07	**0.001**	23%

Bold number means statistically significant (*p* < 0.05).

### 3.6 Meta-regression

The meta-regression analysis for the two polymorphisms, *rs2075650* and *rs157580*, with sufficient studies and for the association of these polymorphisms and the risk of AD, reveals several important findings (Table 4). For *rs2075650*, the publication year is a significant predictor in most genetic models, showing a positive coefficient, indicating that the effect size increases with more recent publications. Sample size and quality score are not significant predictors for this polymorphism. For *rs157580*, the publication year shows a significant negative coefficient in several genetic models, suggesting a decrease in effect size with more recent publications. The quality score is a significant positive predictor for this polymorphism in multiple models, indicating that studies with higher quality scores tend to report stronger associations. Sample size does not significantly predict the effect size for *rs157580*. Overall, the analysis highlights the influence of publication year and quality score on the reported associations for these polymorphisms, with *rs2075650* showing increasing effect sizes over time, and *rs157580* showing stronger associations in higher-quality studies and decreasing effect sizes over time. These analyses allowed identification of methodological quality and publication year as contributors to between-study heterogeneity.

**Table 4:** Meta-regression analysis for two polymorphisms.

Polymorphism	Genetic model	Variable	Coefficient	95%CI		Z-value	*p*-value
				Min.	Max.		
*rs2075650*	G vs. A	Publication year	0.0014	0.0001	0.0027	2.05	**0.0402**
		Sample size	−0.0001	−0.0004	0.0001	−1.23	0.2195
		Quality score	−0.2561	−0.5819	0.0696	−1.54	0.1233
	GG vs. AA	Publication year	0.0024	0.0003	0.0045	2.29	**0.0221**
		Sample size	−0.0001	−0.0005	0.0003	−0.59	0.5550
		Quality score	−0.4507	−0.9896	0.0882	−1.64	0.1012
	AG vs. AA	Publication year	0.0010	−0.0004	0.0024	1.42	0.1544
		Sample size	−0.0002	−0.0004	0.0001	−1.42	0.1557
		Quality score	−0.1478	−0.4994	0.2039	−0.82	0.4102
	GG + AG vs. AA	Publication year	0.0015	0.0002	0.0028	2.34	**0.0192**
		Sample size	−0.0002	−0.0004	0.0001	−1.48	0.1387
		Quality score	−0.2801	−0.5993	0.0392	−1.72	0.0855
	GG vs. AA + AG	Publication year	0.0018	−< 0.0001	0.0036	1.92	0.0549
		Sample size	−< 0.0001	−0.0004	0.0003	−0.28	0.7804
		Quality score	−0.3060	−0.7747	0.1628	−1.28	0.2007
*rs157580*	G vs. A	Publication year	−0.0004	−0.0008	−0.0001	−2.82	**0.0048**
		Sample size	−0.0001	−0.0003	−0.0001	−0.88	0.3773
		Quality score	0.0778	0.0077	0.1478	2.18	**0.0296**
	GG vs. AA	Publication year	−0.0011	−0.0018	−0.0004	−3.14	**0.0017**
		Sample size	−0.0002	−0.0007	0.0002	−1.02	0.3055
		Quality score	0.2193	0.0575	0.3810	2.66	**0.0079**
	AG vs. AA	Publication year	−0.0006	−0.0011	−0.0001	−2.39	**0.0169**
		Sample size	< 0.0001	−0.0003	0.0004	0.25	0.8004
		Quality score	0.1127	−0.0034	0.2288	1.90	0.0570
	GG + AG vs. AA	Publication year	−0.0006	−0.0010	−0.0001	−2.41	**0.0162**
		Sample size	−< 0.0001	−0.0003	0.0002	−0.30	0.7650
		Quality score	0.0919	−0.0094	0.1932	1.78	0.0755
	GG vs. AA + AG	Publication year	−0.0008	−0.0014	−0.0001	−2.38	**0.0171**
		Sample size	−0.0002	−0.0007	0.0002	−1.11	0.2666
		Quality score	0.1616	0.0084	0.3149	2.07	**0.0387**

Bold number means statistically significant (*p* < 0.05).

### 3.7 Sensitivity analysis

The stability of the pooled ORs for the association of *rs2075650, rs157580, rs8106922,* and *rs157581* polymorphisms and the risk of AD, as indicated by both one-study-removed and cumulative analyses, suggests that the overall effect estimates for these polymorphisms are robust. This means that removing any single study or adding studies sequentially did not significantly alter the combined ORs, further strengthening the confidence in the reported associations. This consistency implies that the observed relationships between these polymorphisms and the studied condition are reliable and not unduly influenced by individual studies.

### 3.8 TSA

The TSA plots for the association of *rs2075650* and *rs157580* polymorphisms and the risk of AD, as shown in Supplementary Figures S1–S10, provide important insights into the strength and reliability of the evidence. For *rs2075650*, the fact that the z-curve crossed the RIS lines in all genetic models indicates that the cumulative evidence is sufficient and that the observed associations are likely reliable and not due to random chance. This strengthens the conclusion that *rs2075650* is significantly associated with the studied condition across various genetic models. For *rs157580*, the z-curve crossing the RIS lines in the homozygous and heterozygous models suggests that the evidence for these specific genetic models is also sufficient and reliable. However, the z-curve not crossing the RIS lines in other models implies that more studies might be needed to reach a definitive conclusion for those models. Overall, these TSA results add robustness to the findings for *rs2075650* and provide specific evidence for the homozygous and heterozygous models of *rs157580*, indicating solid and trustworthy associations in those contexts.

### 3.9 Publication bias

**Figure 2:**
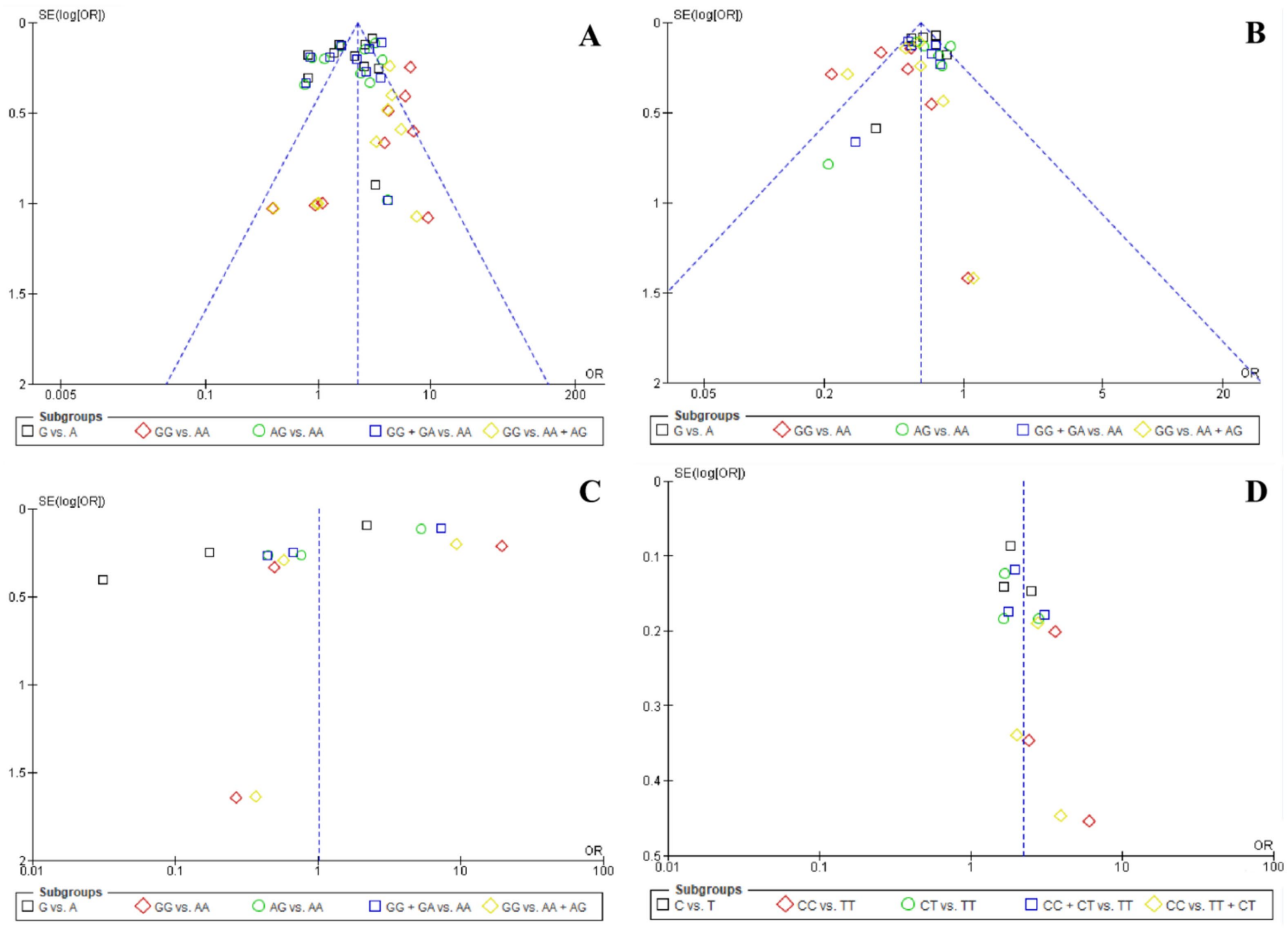
Funnel plot analysis of the association of four polymorphisms and the risk of Alzheimer’s disease in five genetic models: **(A)** *rs2075650*, **(B)** *rs157580*, **(C)** *rs8106922*, **(D)** *rs157581.*

The funnel plots for *the* association of *rs2075650, rs157580, rs8106922,* and *rs157581* polymorphisms and the risk of AD, as shown in Figure 2, reveal some important observations about publication bias. Specifically, for the homozygous and recessive models of *rs2075650*, both Egger’s and Begg’s tests indicated significant bias. This suggests that the observed associations in these models may be influenced by factors such as selective reporting or publication bias, which could affect the reliability of the findings for *rs2075650* in these specific genetic models. For the other polymorphisms and models, no significant bias was detected, indicating that the associations reported in those cases are less likely to be affected by publication bias.

### 3.10 Functional and mechanistic insights for significant TOMM40 polymorphisms

**Figure 3:**
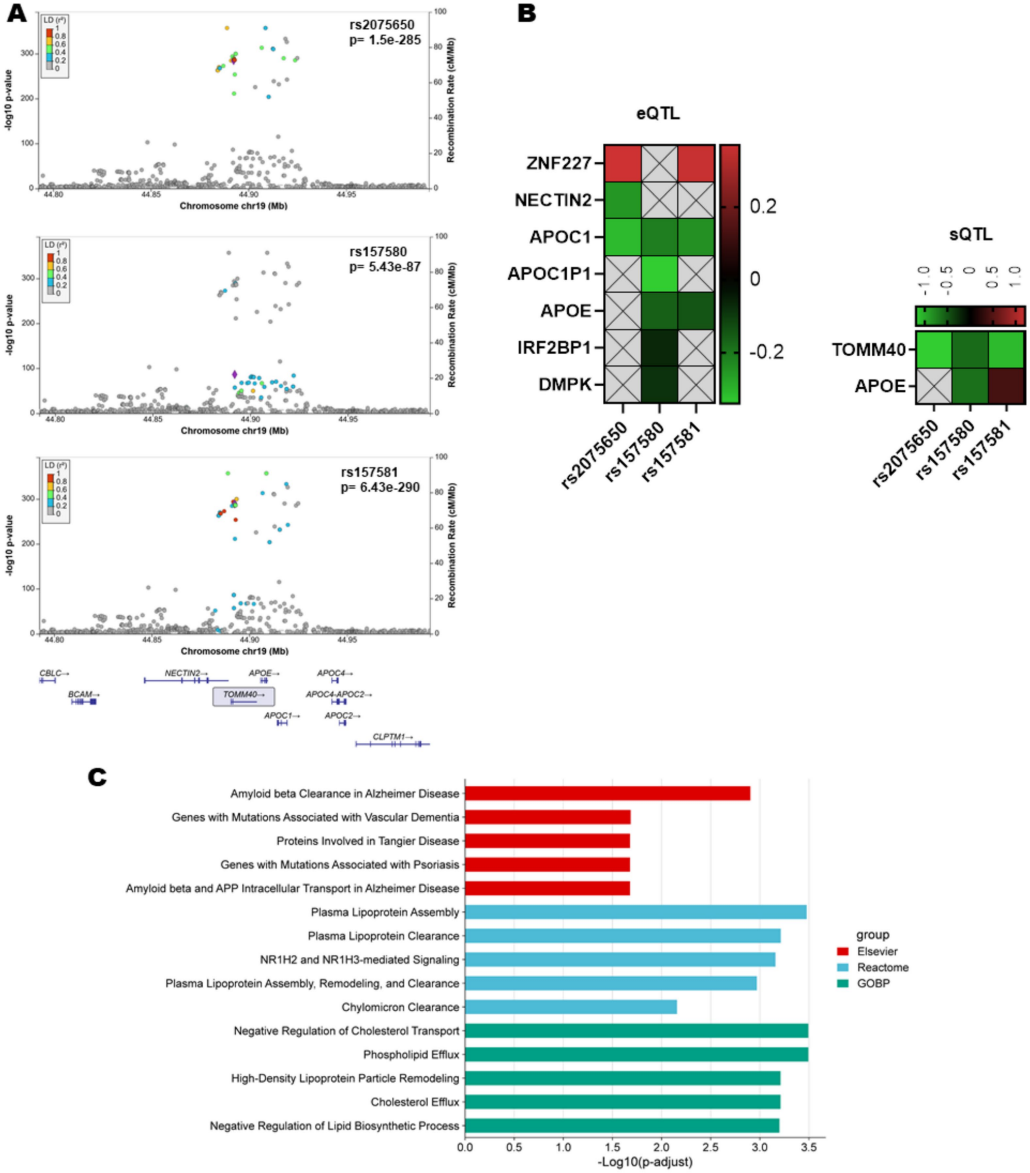
Functional characterization of *TOMM40* polymorphisms associated with Alzheimer’s disease. **(A)** LocusZoom plots from the IGAP Stage 1 dataset showing regional association patterns for rs2075650, rs157580, and rs157581. rs2075650 and rs157581 fall within the APOE–TOMM40–APOC1 LD block, whereas rs157580 shows minimal LD with surrounding variants. **(B)** Heatmaps of significant eQTL (left) and sQTL (right) associations (*p* < 0.05) from GTEx v8, visualized using normalized effect size (NES) values. rs2075650 and rs157581 show strong sQTL effects on TOMM40 splicing, whereas rs157580 exhibits broader eQTL influences across multiple genes. **(C)** Pathway enrichment for eQTL-associated genes (≥3 targets). rs157580-related genes were enriched in lipid and amyloid pathways, suggesting a mechanism distinct from the splicing effects driving rs2075650 and rs157581. AD, Alzheimer’s disease; LD, linkage disequilibrium; eQTL, expression quantitative trait locus; sQTL, splicing quantitative trait locus; NES, normalized effect size.

To investigate potential mechanisms underlying the three polymorphisms significantly associated with AD in the meta-analysis (*rs2075650*, *rs157580*, and *rs157581*), we integrated fine-mapping, QTL analyses, and pathway enrichment (Figures 3A–C). LocusZoom plots from the IGAP Stage 1 dataset showed that *rs2075650* and *rs157581* lie within a dense LD block spanning the *APOE–TOMM40–APOC1* region, whereas *rs157580* exhibits minimal LD with surrounding variants, indicating a more independent signal (Figure 3A). Consistent with this structure, eQTL analysis revealed that *rs157580* had the broadest transcriptional footprint, showing significant associations with the expression of *APOC1P1*, *APOE*, *IRF2BP1*, *DMPK*, and *APOC1*, while *rs2075650* and *rs157581* were associated with a more restricted set of genes (Figure 3B). In contrast, sQTL analysis showed that rs2075650 and rs157581 were the variants most strongly linked to altered *TOMM40* splicing, whereas *rs157580* exhibited a weaker splicing effect. Pathway enrichment of genes significantly associated with *rs157580* expression highlighted AD–related amyloid pathways, plasma lipoprotein assembly and clearance, and cholesterol transport processes (Figure 3C). Integrating these findings, *rs2075650* and *rs157581*—both associated with increased AD risk—are characterized by strong *TOMM40* splicing alterations and localization within the major LD block of the *APOE–TOMM40–APOC1* locus, whereas the protective variant *rs157580* shows an LD-independent profile marked by transcriptional effects on lipid- and amyloid-related genes. Together, these results indicate that although all three SNPs map to the same genomic region, the two risk variants are characterized by strong *TOMM40* splicing perturbation and localization within the major LD block of the *APOE–TOMM40–APOC1* locus, whereas the protective variant represents an LD-independent signal associated with distinct transcriptional pathways involving lipid and amyloid biology.

## 4 Discussion

The meta-analysis identifies significant associations between specific *TOMM40* polymorphisms and AD risk. *rs2075650* and *rs157581* are linked to elevated susceptibility, while *rs157580* shows a protective effect, with findings most robust for *rs2075650* in Caucasian populations and larger studies. However, *rs8106922*, *rs11556505*, and *rs1160985* show no significant associations. The meta-regression analysis highlights the influence of publication year and study quality, with *rs2075650* showing increasing effect sizes over time. Despite these consistent results, significant publication bias in some *rs2075650* models suggests potential selective reporting. Importantly, integrative functional analyses revealed that *rs2075650* and *rs157581* act within the APOE-linked LD block and influence *TOMM40* splicing, whereas *rs157580* shows an LD-independent regulatory profile affecting the expression of genes involved in lipid and amyloid pathways. These combined findings emphasize both the statistical and biological relevance of *TOMM40* variants in AD susceptibility.

The etiology of common LOAD is only partly understood and appears to be extremely complex, involving numerous genetic and environmental factors. The most significant environmental risk factor for developing LOAD is aging itself. Both aging and LOAD are strongly associated with mitochondrial dysfunction and increased oxidative stress (Anandatheerthavarada et al., 2003; Wang et al., 2014). While much is known about the clinical manifestations of AD (a neurodegenerative disorder), the etiology and pathophysiology remain to be fully explored. Mitochondrial dysfunction is increasingly recognized in both pathological and non-pathological aging, and *TOMM40* may play a significant role in this area by influencing mitochondrial neurotoxicity (Ferencz et al., 2013; Bekris et al., 2012; Bis et al., 2012). *Tom40*, a subunit of the translocase of the outer membrane (*TOM*), is encoded by the *TOMM40* gene. This gene facilitates the passage of cytoplasmic peptides and proteins, leading to the accumulation of amyloid precursor protein (Roses et al., 2010). *TOMM40* is thought to be involved in the translocation and metabolism of APP/Aβ and the regulation of APOE. It is plausible that *TOMM40* plays a role in AD through its effects on mitochondrial function. Consequently, *TOMM40* expression levels may influence mitochondrial function and contribute to AD risk (Lee et al., 2021; Di Stolfo et al., 2024). Our integrative analyses support these biological roles by showing that the risk variants rs2075650 and rs157581 exert strong sQTL effects on *TOMM40*, suggesting altered mitochondrial protein import through disrupted splicing. Their location within the APOE–*TOMM40*–APOC1 LD block further indicates coordinated regulatory changes affecting lipid and amyloid pathways. In contrast, the protective rs157580 variant exhibits an LD-independent profile and influences the expression of genes involved in lipid and amyloid biology, consistent with a distinct protective mechanism.

The presence of unfavorable alleles in the *APOE cluster, TOMM40,* or *APOC1* is known to facilitate the onset of dementia under oxidative stress (Prendecki et al., 2018). A systematic review (Chen et al., 2022) identified multiple *TOMM40* polymorphisms potentially associated with healthy aging. AD is the primary cause of dementia, and age is its major risk factor (Harman, 2006). Amyloid plaques and neurofibrillary tangles (NFTs), the two best-known pathological hallmarks of AD, are observed in the aging human brain, even in individuals without dementia (Sengoku, 2020). These findings also have potential clinical implications. Genetic markers such as rs2075650 and rs157581 may help identify individuals at higher risk for AD, enabling earlier intervention strategies and personalized monitoring. Furthermore, clarifying the regulatory functions of these variants may inform therapeutic approaches targeting mitochondrial function, lipid handling, or amyloid biology.

Although bioinformatics and association analyses provide important mechanistic insights into how *TOMM40* polymorphisms may influence alternative splicing and gene expression, these findings remain inferential. Functional validation using experimental cellular and molecular models will be necessary to confirm causality and further elucidate the biological mechanisms underlying AD susceptibility.

Despite robust findings, several limitations should be noted. Significant publication bias in the homozygous and recessive models of *rs2075650* suggests that these associations may be influenced by selective reporting. High heterogeneity across analyses likely reflects differences in study design, population demographics, or other factors. For certain polymorphisms, such as *rs1160985* and *rs11556505*, only a few studies with relatively small sample sizes were available. This limited data reduces statistical power, affects the robustness of conclusions, and restricts generalizability. Our focus on predominantly Caucasian and Asian populations may further limit applicability to other ethnic groups. Additionally, potential confounding factors—including gene interactions, environmental influences, and comorbidities—could not be evaluated due to insufficient and heterogeneous reporting. Importantly, mechanistic insights from bioinformatics analyses—such as effects on *TOMM40* splicing and gene expression—are inferential and do not establish causality; experimental validation in cellular or animal models is required. Future studies with larger, well-powered cohorts and standardized reporting are needed to confirm these findings, clarify the impact of confounding factors, and strengthen observed associations.

## 5 Conclusion

This study demonstrates that *rs2075650* and *rs157581* are associated with increased AD risk, whereas rs157580 shows a consistent protective effect. Integrating genome-wide, regulatory, and functional evidence reveals that the risk variants likely influence AD susceptibility through *TOMM40* splicing disruptions within the APOE-linked LD block, while the protective variant appears to act independently by modulating lipid- and amyloid-related gene expression. These findings strengthen the evidence for *TOMM40* as a biologically meaningful contributor to AD risk and suggest that these polymorphisms could serve as genetic biomarkers for risk stratification in clinical or research settings. Understanding the functional impact of these variants may also inform future therapeutic strategies targeting *TOMM40*-mediated pathways, particularly those related to mitochondrial function, lipid metabolism, and amyloid processing. Future research should include experimental validation of splicing and expression effects, exploration of gene–environment and gene–gene interactions, and larger multi-ethnic cohort studies to confirm these associations and assess population-specific effects. Such studies will help clarify the translational potential of *TOMM40* polymorphisms in AD prevention, diagnosis, and therapy.

## Data Availability

The original contributions presented in the study are included in the article/Supplementary material, further inquiries can be directed to the corresponding author.
