# Supplementary material for: Functional and genetic divergence of aging-related TOMM40 polymorphisms in Alzheimer’s disease: an integrative bioinformatics and systematic review with meta-analysis and trial sequential analysis
Source: Front Neurosci. 2026 Apr 10;20:1772368. doi: 10.3389/fnins.2026.1772368 (PMC13106414; doi:10.3389/fnins.2026.1772368)
Supplement: Supplementary file 1 [file Data_sheet_1.docx]

**
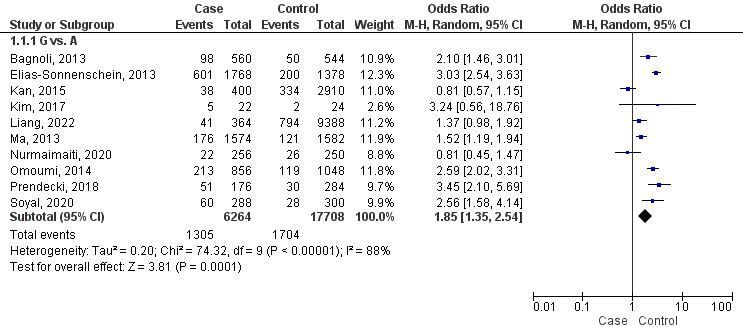
**

**Supplemental Digital Content 1. Figure S1**: Forest plot analysis of association of *rs2075650* polymorphism and the risk of Alzheimer's disease in allelic model.

**
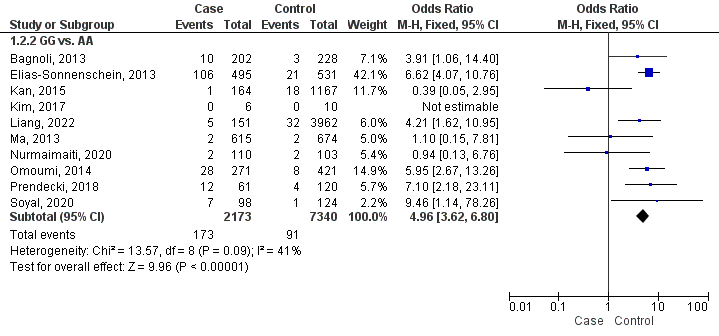
**

**Supplemental Digital Content 1. Figure S2**: Forest plot analysis of association of *rs2075650* polymorphism and the risk of Alzheimer's disease in homozygous model.

**
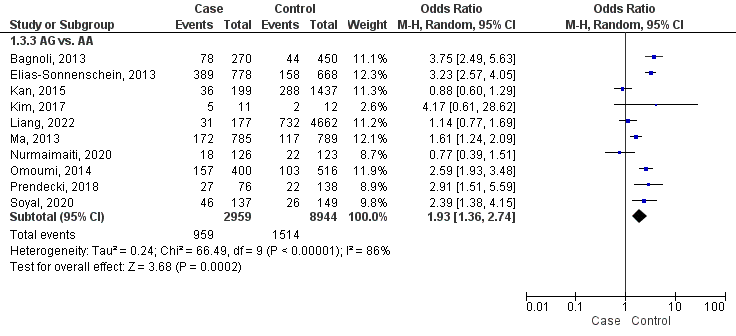
**

**Supplemental Digital Content 1. Figure S3**: Forest plot analysis of association of *rs2075650* polymorphism and the risk of Alzheimer's disease in heterozygous model.

**
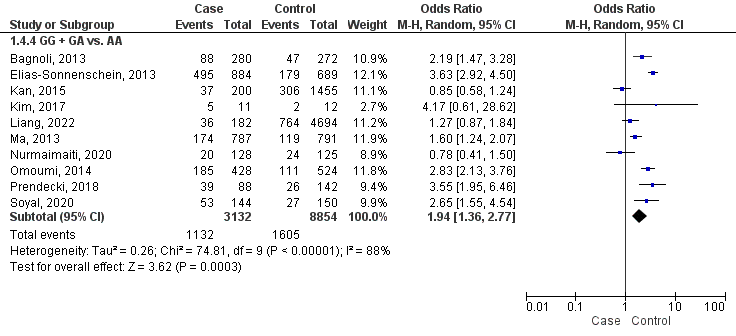
**

**Supplemental Digital Content 1. Figure S4**: Forest plot analysis of association of *rs2075650* polymorphism and the risk of Alzheimer's disease in dominant model.

**
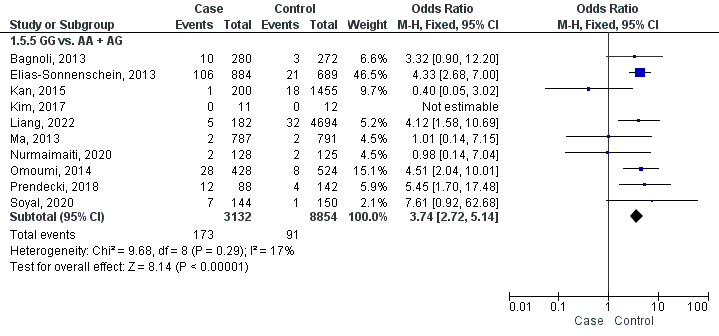
**

**Supplemental Digital Content 1. Figure S5**: Forest plot analysis of association of *rs2075650* polymorphism and the risk of Alzheimer's disease in recessive model.

**
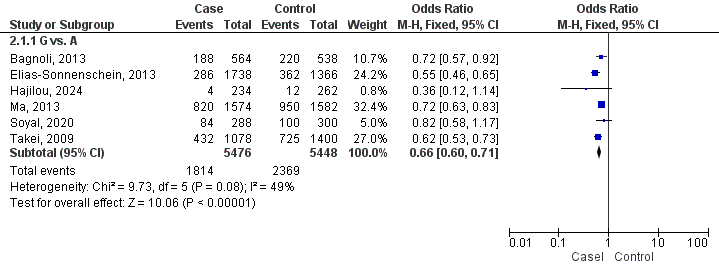
**

**Supplemental Digital Content 1. Figure S6**: Forest plot analysis of association of *rs157580* polymorphism and the risk of Alzheimer's disease in allelic model.

**
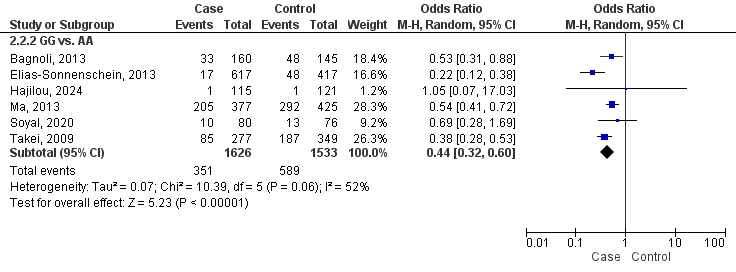
**

**Supplemental Digital Content 1. Figure S7**: Forest plot analysis of association of *rs157580* polymorphism and the risk of Alzheimer's disease in homozygous model.

**
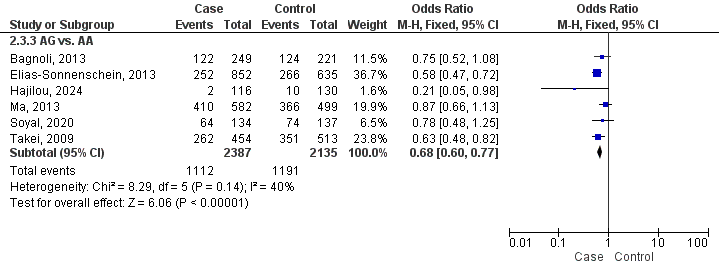
**

**Supplemental Digital Content 1. Figure S8**: Forest plot analysis of association of *rs157580* polymorphism and the risk of Alzheimer's disease in heterozygous model.

**
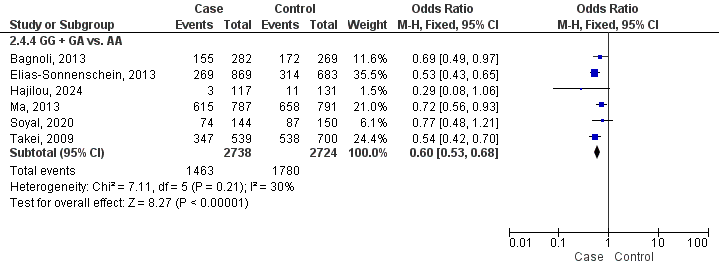
**

**Supplemental Digital Content 1. Figure S9**: Forest plot analysis of association of *rs157580* polymorphism and the risk of Alzheimer's disease in dominant model.

**
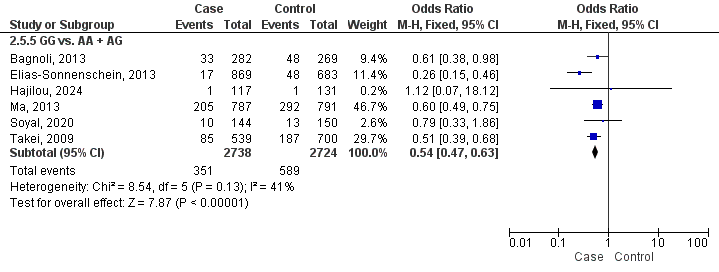
**

**Supplemental Digital Content 1. Figure S10**: Forest plot analysis of association of *rs157580* polymorphism and the risk of Alzheimer's disease in recessive model.

**

**

**Supplemental Digital Content 1. Figure S11**: Forest plot analysis of association of *rs8106922* polymorphism and the risk of Alzheimer's disease in allelic model.

**
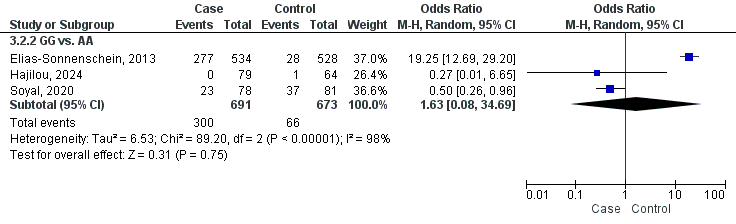
**

**Supplemental Digital Content 1. Figure S12**: Forest plot analysis of association of *rs8106922* polymorphism and the risk of Alzheimer's disease in homozygous model.

**
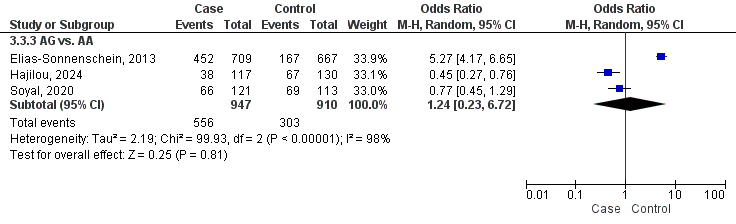
**

**Supplemental Digital Content 1. Figure S13**: Forest plot analysis of association of *rs8106922* polymorphism and the risk of Alzheimer's disease in heterozygous model.

**
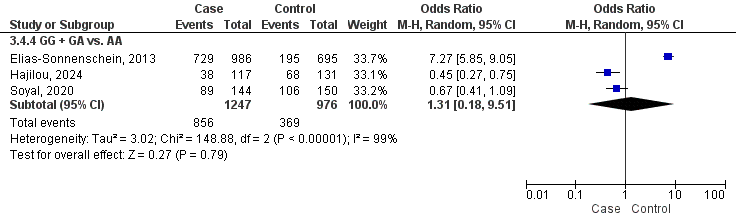
**

**Supplemental Digital Content 1. Figure S14**: Forest plot analysis of association of *rs8106922* polymorphism and the risk of Alzheimer's disease in dominant model.

**
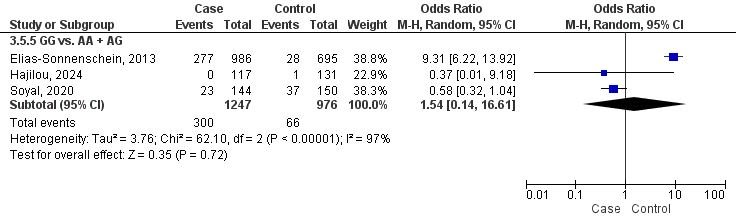
**

**Supplemental Digital Content 1. Figure S15**: Forest plot analysis of association of *rs8106922* polymorphism and the risk of Alzheimer's disease in recessive model.

**
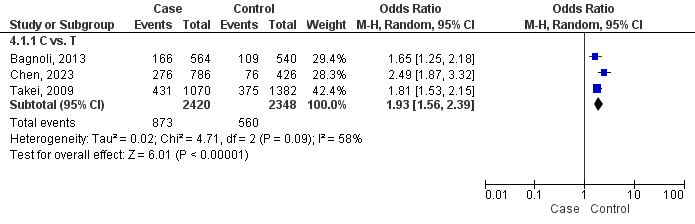
**

**Supplemental Digital Content 1. Figure S16**: Forest plot analysis of association of *rs157581* polymorphism and the risk of Alzheimer's disease in allelic model.

**
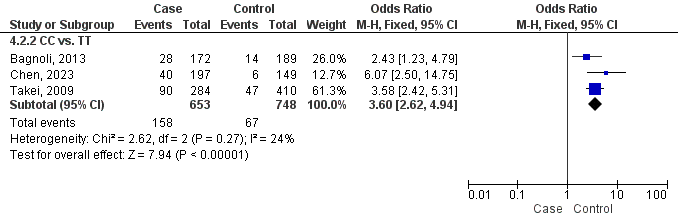
**

**Supplemental Digital Content 1. Figure S17**: Forest plot analysis of association of *rs157581* polymorphism and the risk of Alzheimer's disease in homozygous model.

**
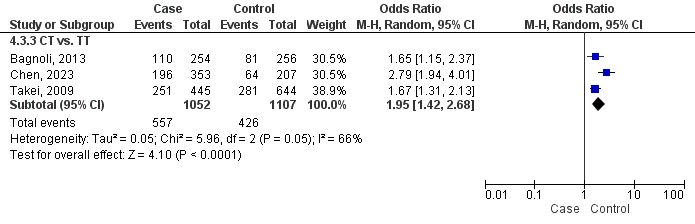
**

**Supplemental Digital Content 1. Figure S18**: Forest plot analysis of association of *rs157581* polymorphism and the risk of Alzheimer's disease in heterozygous model.

**
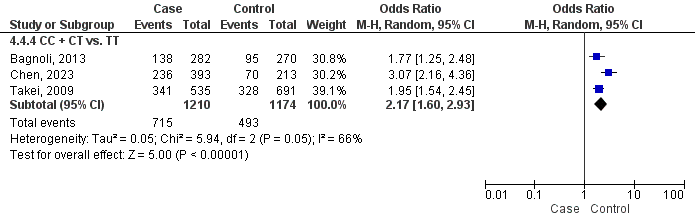
**

**Supplemental Digital Content 1. Figure S19**: Forest plot analysis of association of *rs157581* polymorphism and the risk of Alzheimer's disease in dominant model.

**
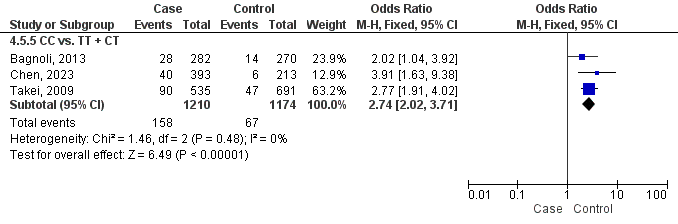
**

**Supplemental Digital Content 1. Figure S20**: Forest plot analysis of association of *rs157581* polymorphism and the risk of Alzheimer's disease in recessive model.

**
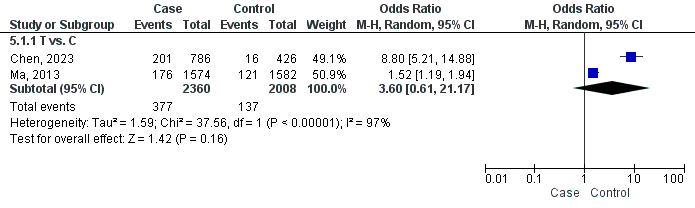
**

**Supplemental Digital Content 1. Figure S21**: Forest plot analysis of association of *rs11556505* polymorphism and the risk of Alzheimer's disease in allelic model.

**
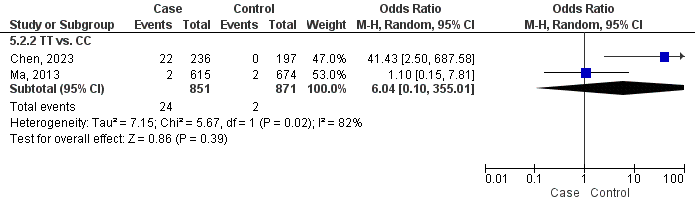
**

**Supplemental Digital Content 1. Figure S22**: Forest plot analysis of association of *rs11556505* polymorphism and the risk of Alzheimer's disease in homozygous model.

**
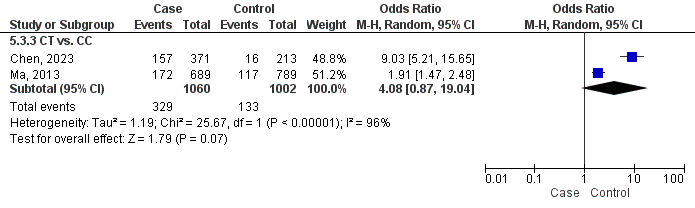
**

**Supplemental Digital Content 1. Figure S23**: Forest plot analysis of association of *rs11556505* polymorphism and the risk of Alzheimer's disease in heterozygous model.

**
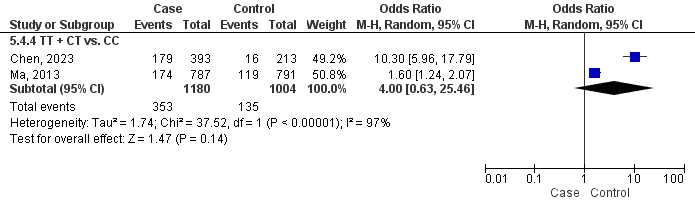
**

**Supplemental Digital Content 1. Figure S24**: Forest plot analysis of association of *rs11556505* polymorphism and the risk of Alzheimer's disease in dominant model.

**
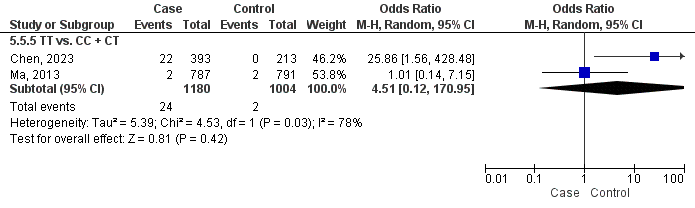
**

**Supplemental Digital Content 1. Figure S25**: Forest plot analysis of association of *rs11556505* polymorphism and the risk of Alzheimer's disease in recessive model.

**
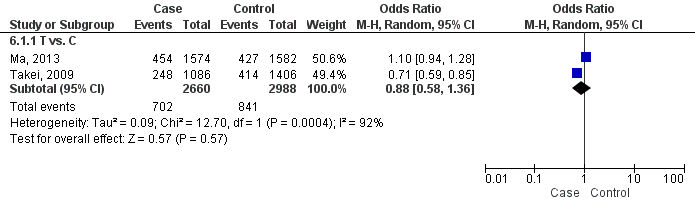
**

**Supplemental Digital Content 1. Figure S26**: Forest plot analysis of association of *rs1160985* polymorphism and the risk of Alzheimer's disease in allelic model.

**
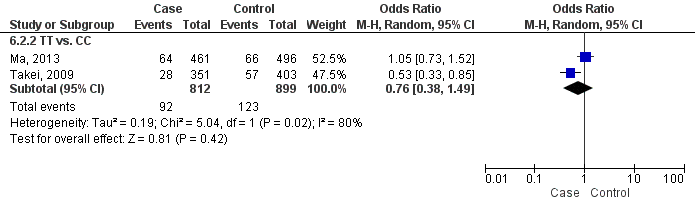
**

**Supplemental Digital Content 1. Figure S27**: Forest plot analysis of association of *rs1160985* polymorphism and the risk of Alzheimer's disease in homozygous model.

**
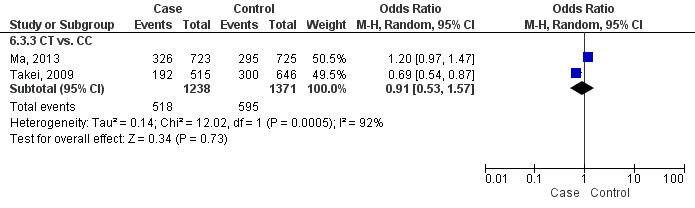
**

**Supplemental Digital Content 1. Figure S28**: Forest plot analysis of association of *rs1160985* polymorphism and the risk of Alzheimer's disease in heterozygous model.

**
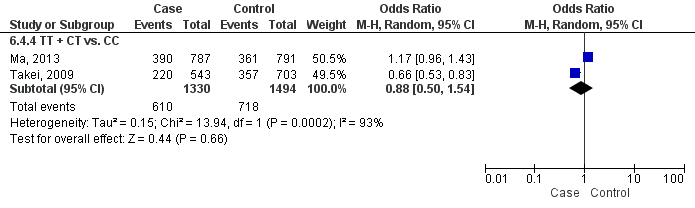
**

**Supplemental Digital Content 1. Figure S29**: Forest plot analysis of association of *rs1160985* polymorphism and the risk of Alzheimer's disease in dominant model.

**
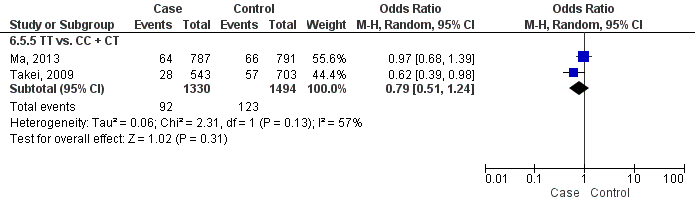
**

**Supplemental Digital Content 1. Figure S30**: Forest plot analysis of association of *rs1160985* polymorphism and the risk of Alzheimer's disease in recessive model.
