# Supplementary material for: Functional and genetic divergence of aging-related TOMM40 polymorphisms in Alzheimer’s disease: an integrative bioinformatics and systematic review with meta-analysis and trial sequential analysis
Source: Front Neurosci. 2026 Apr 10;20:1772368. doi: 10.3389/fnins.2026.1772368 (PMC13106414; doi:10.3389/fnins.2026.1772368)
Supplement: Supplementary file 2 [file Data_sheet_2.docx]

**
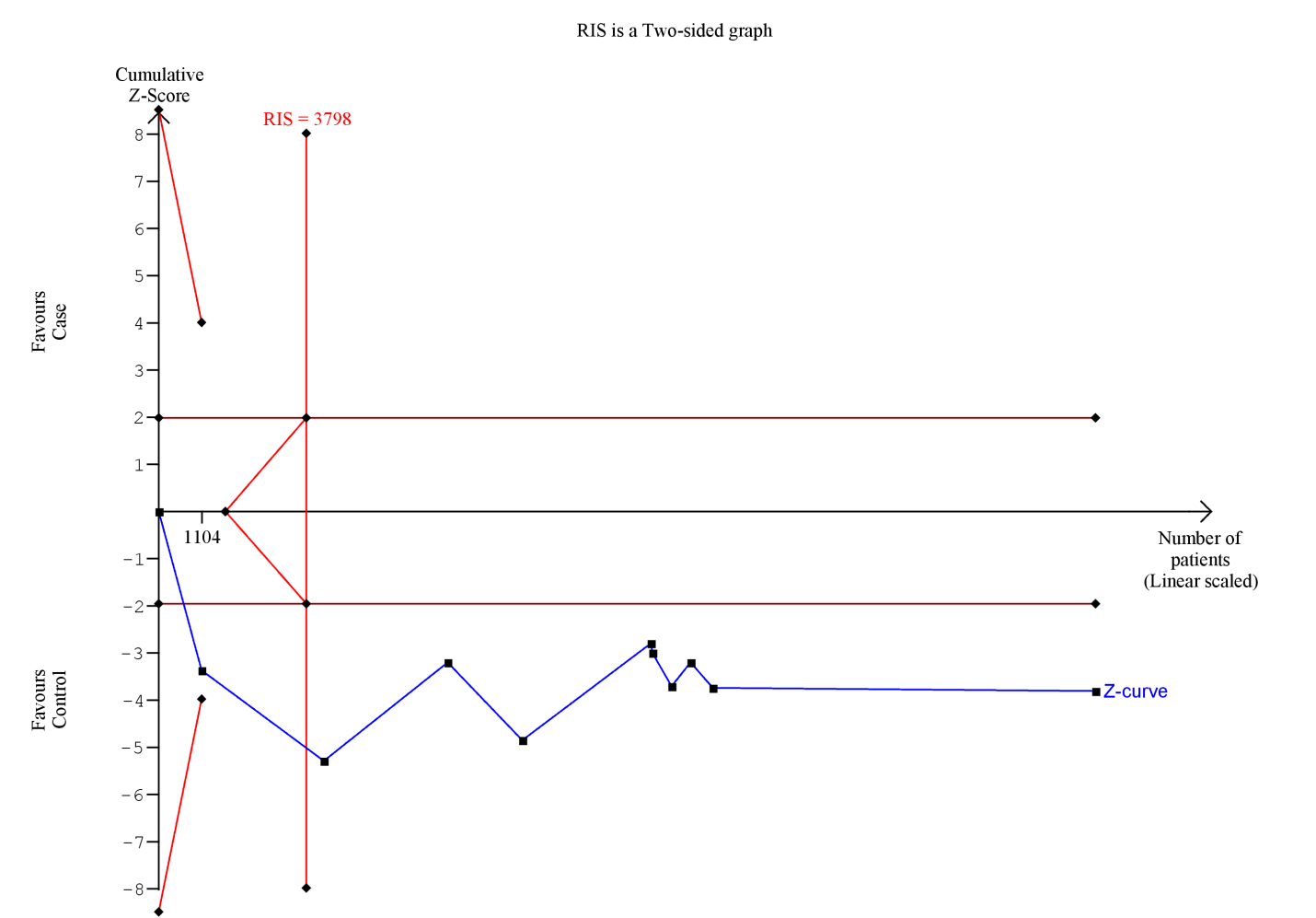
**

**Supplemental Digital Content 2. Figure S1**: Trial sequential analysis of association of *rs2075650* polymorphism and the risk of Alzheimer's disease in allelic model.

**
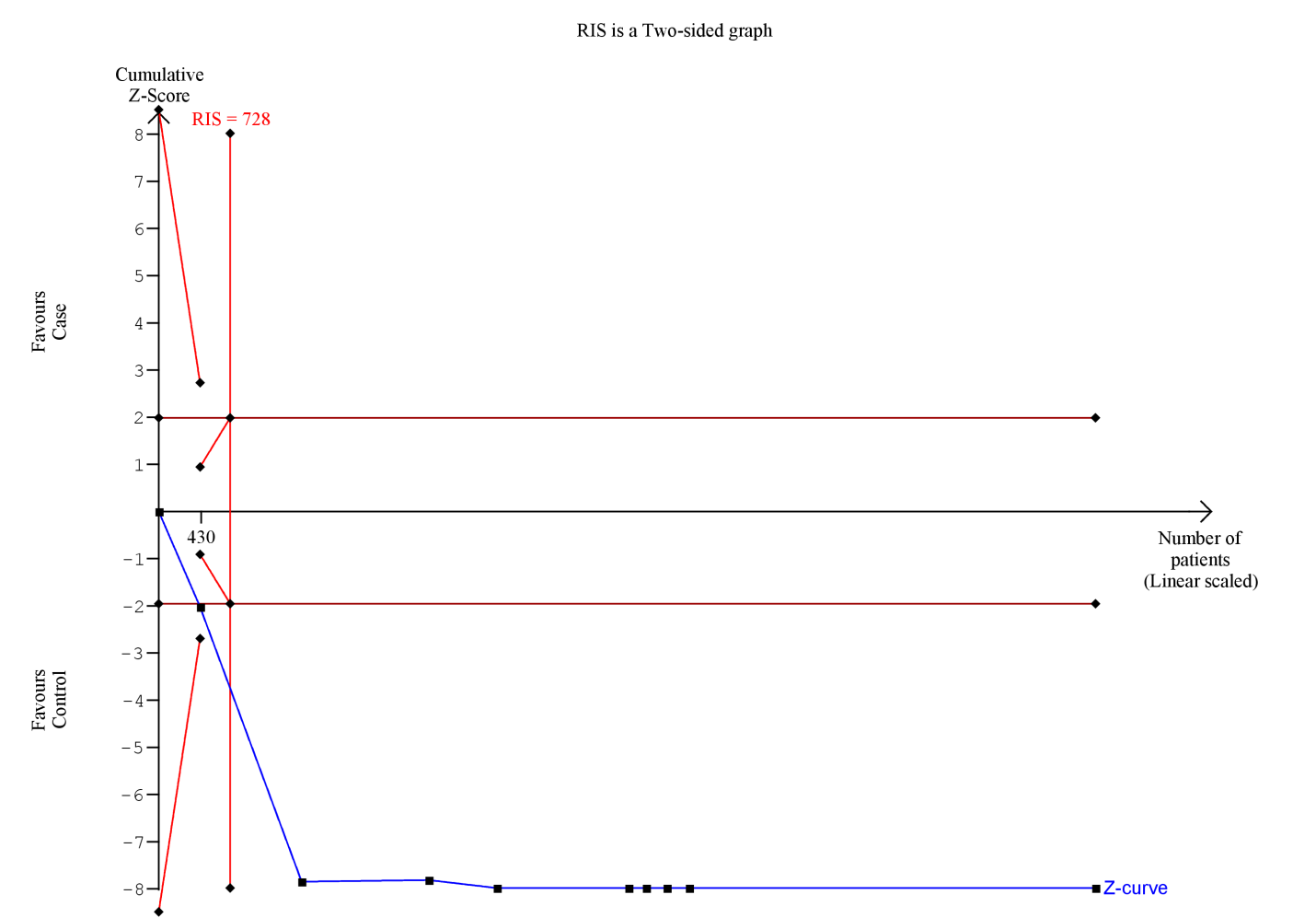
**

**Supplemental Digital Content 2. Figure S2**: Trial sequential analysis of association of *rs2075650* polymorphism and the risk of Alzheimer's disease in homozygous model.

**
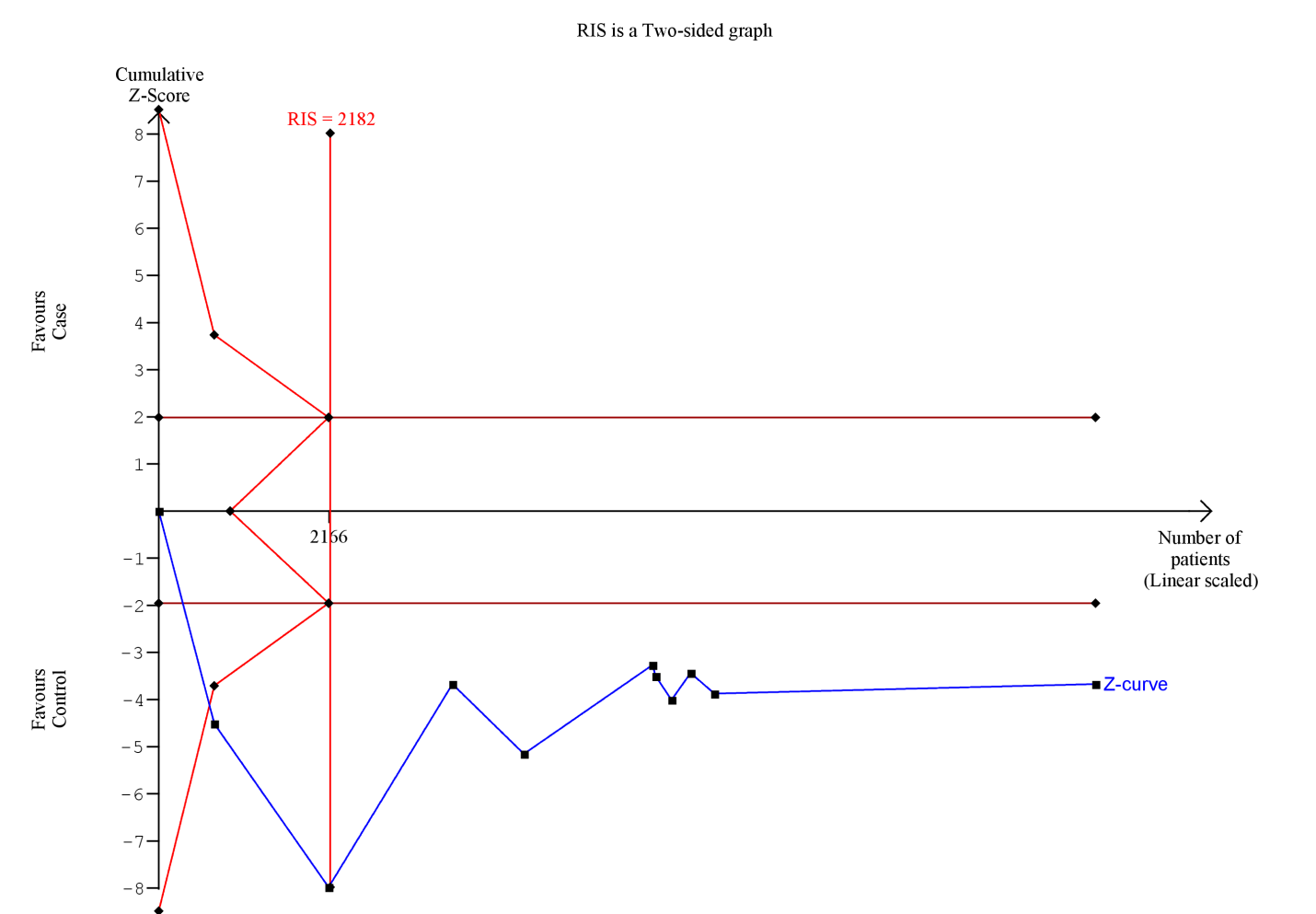
**

**Supplemental Digital Content 2. Figure S3**: Trial sequential analysis of association of *rs2075650* polymorphism and the risk of Alzheimer's disease in heterozygous model.

**
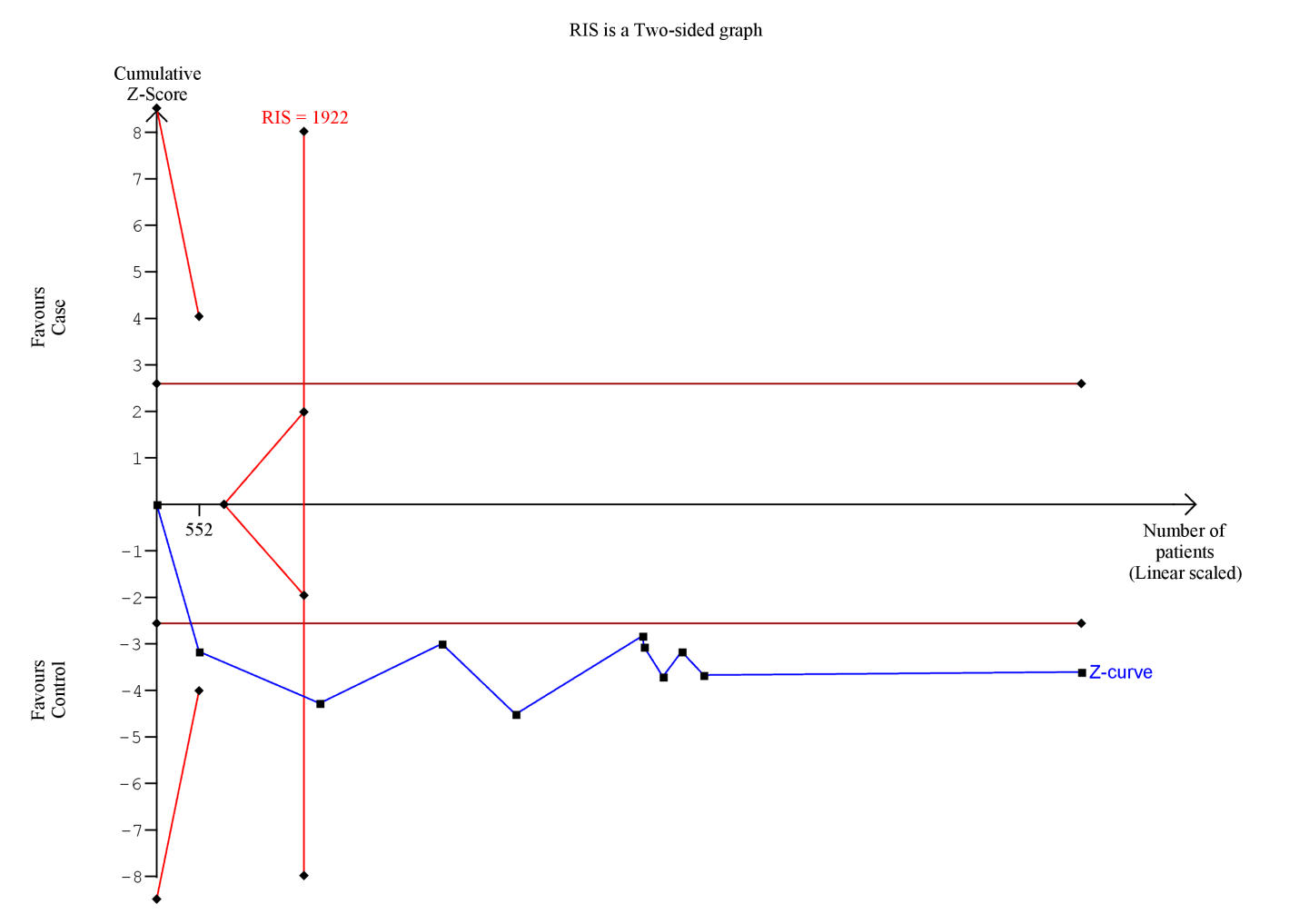
**

**Supplemental Digital Content 2. Figure S4**: Trial sequential analysis of association of *rs2075650* polymorphism and the risk of Alzheimer's disease in dominant model.

**
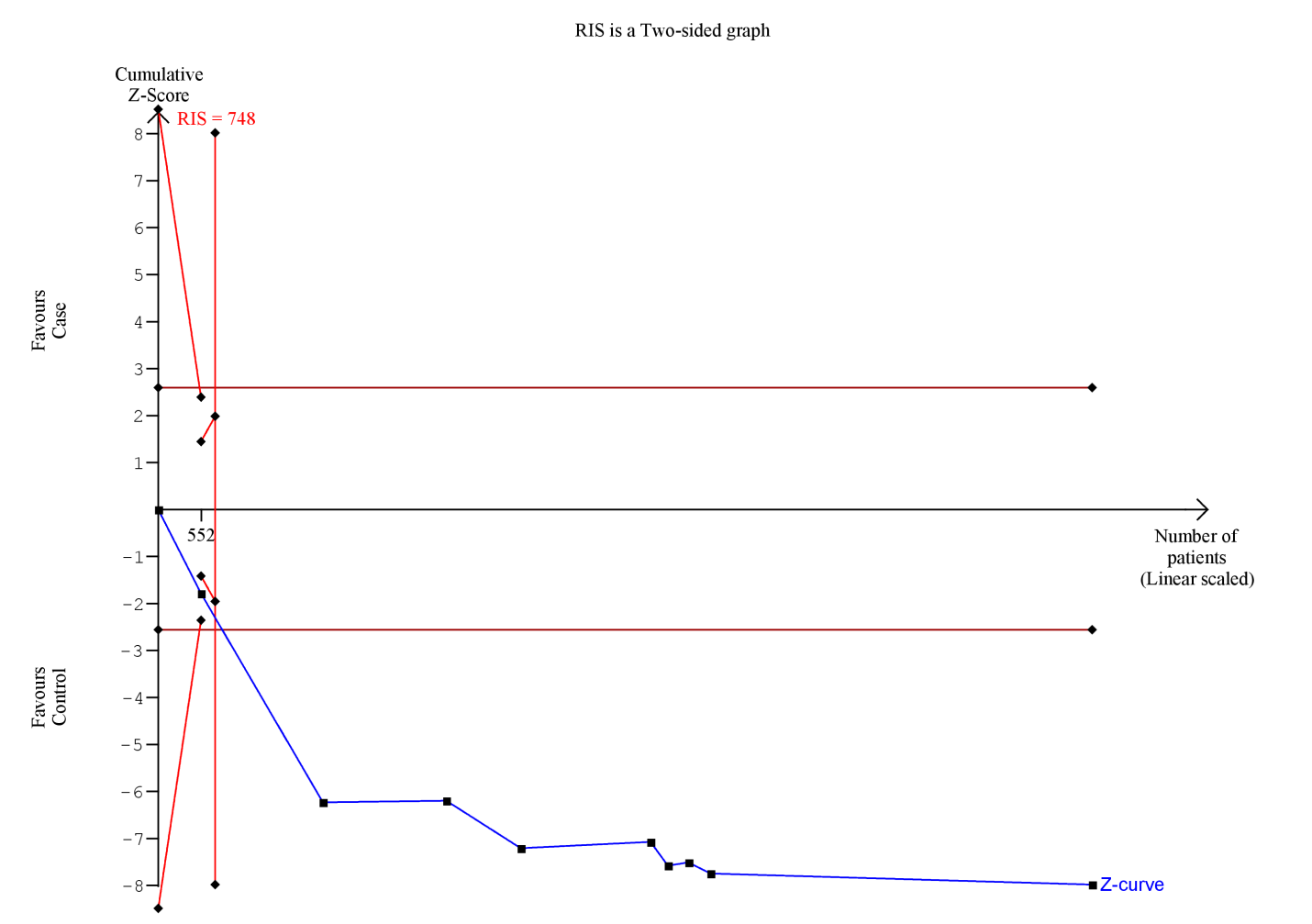
**

**Supplemental Digital Content 2. Figure S5**: Trial sequential analysis of association of *rs2075650* polymorphism and the risk of Alzheimer's disease in recessive model.

**
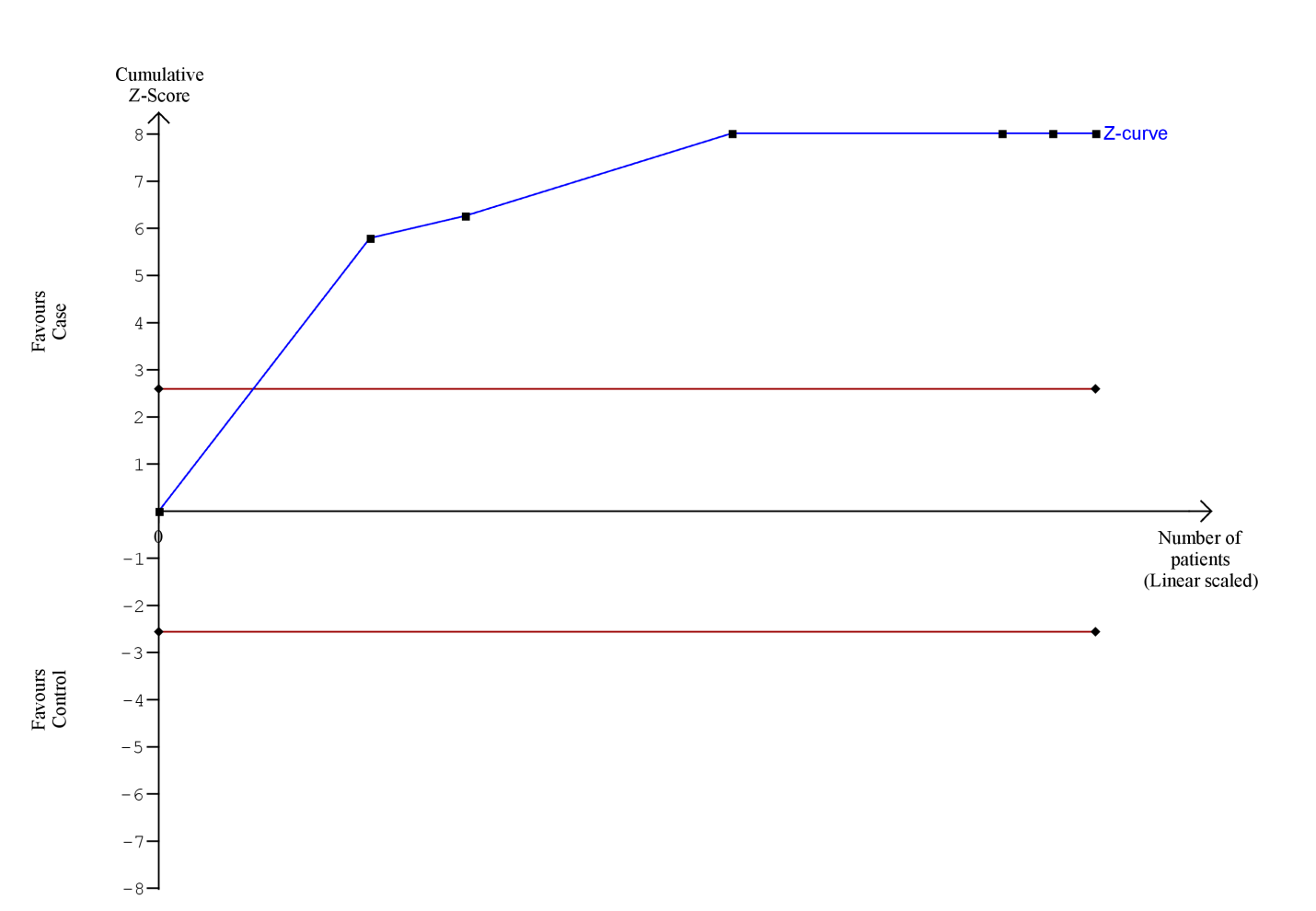

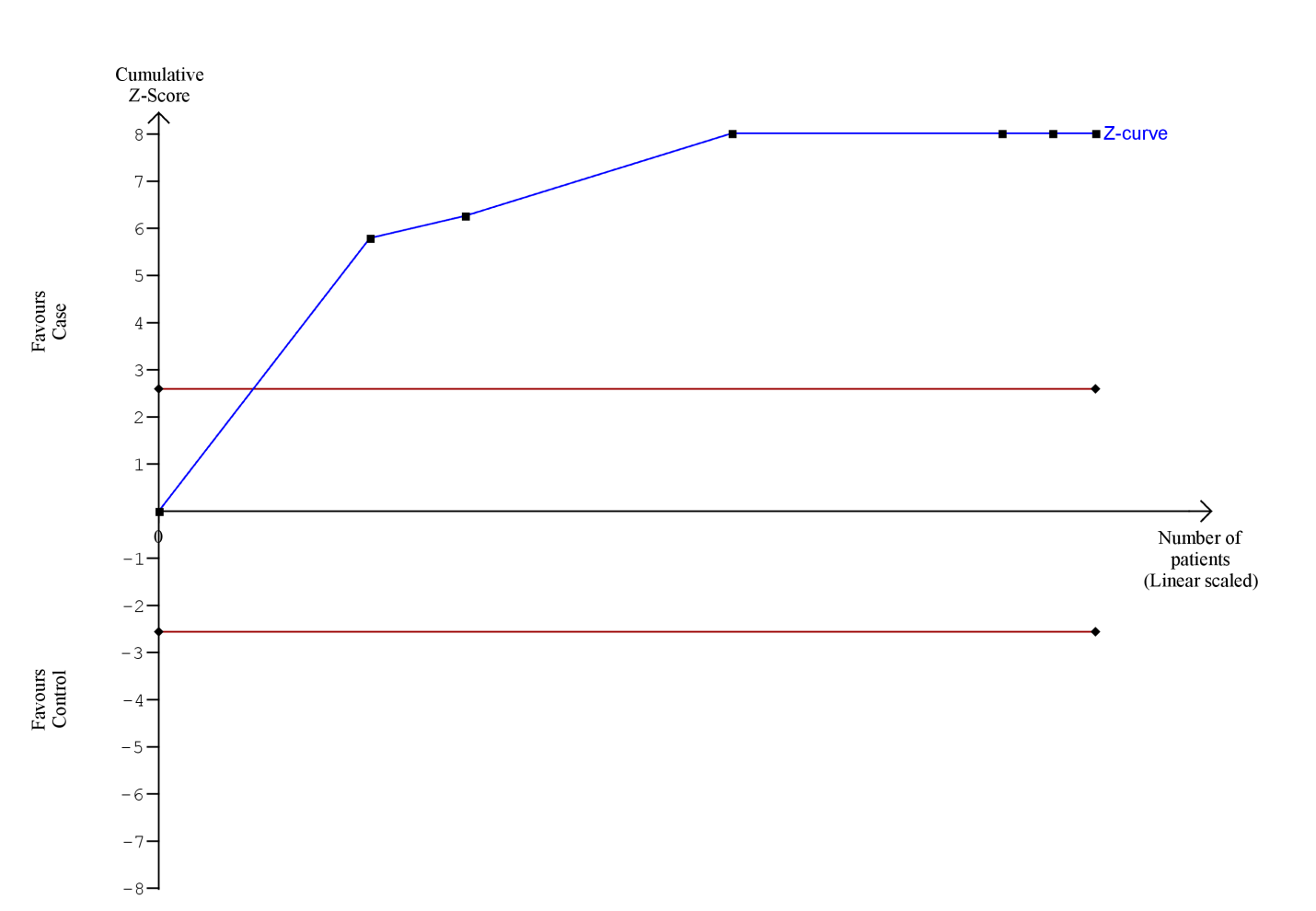
**

**Supplemental Digital Content 2. Figure S6**: Trial sequential analysis of association of *rs157580* polymorphism and the risk of Alzheimer's disease in allelic model.

**
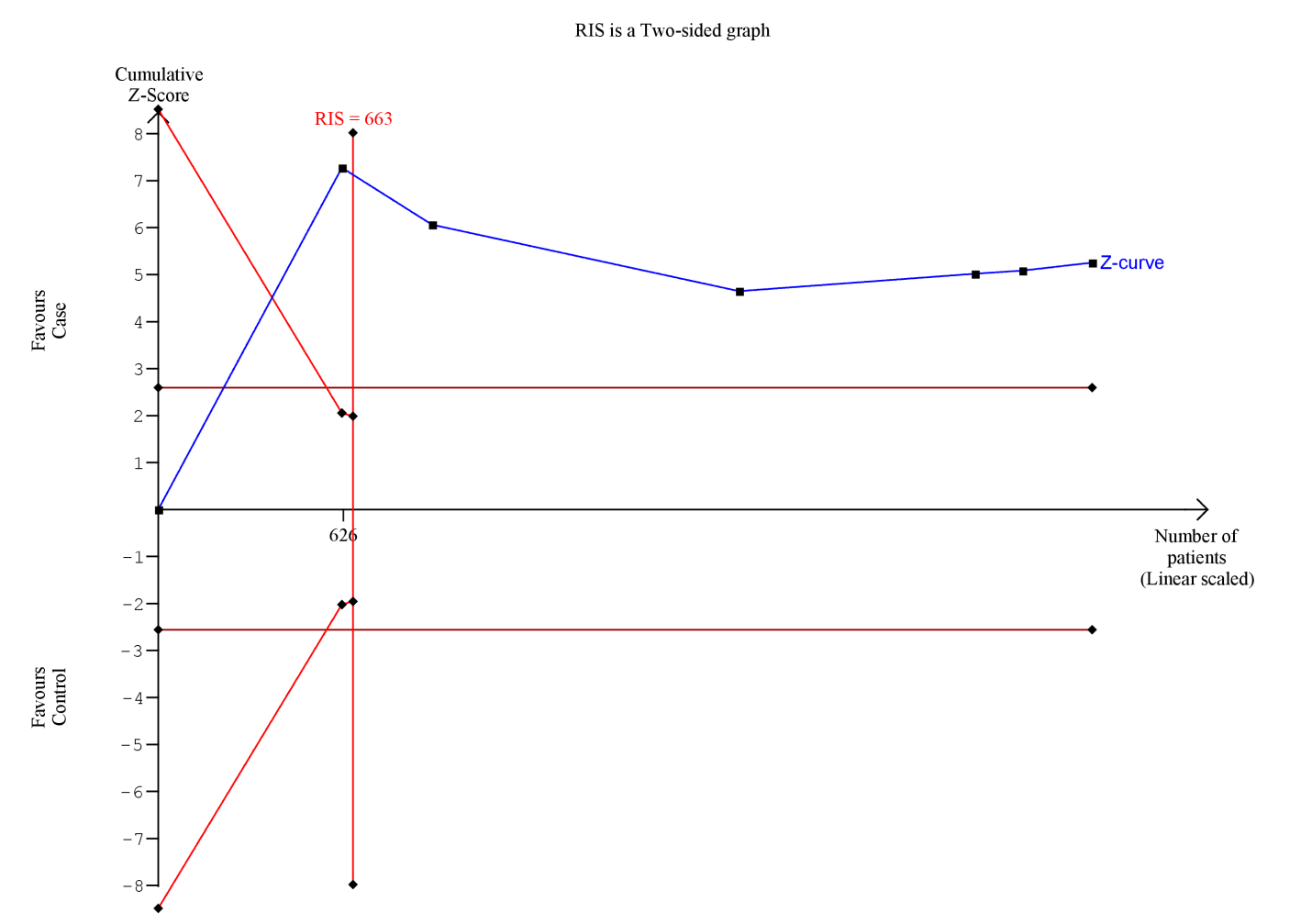
**

**Supplemental Digital Content 2. Figure S7**: Trial sequential analysis of association of *rs157580* polymorphism and the risk of Alzheimer's disease in homozygous model.

**
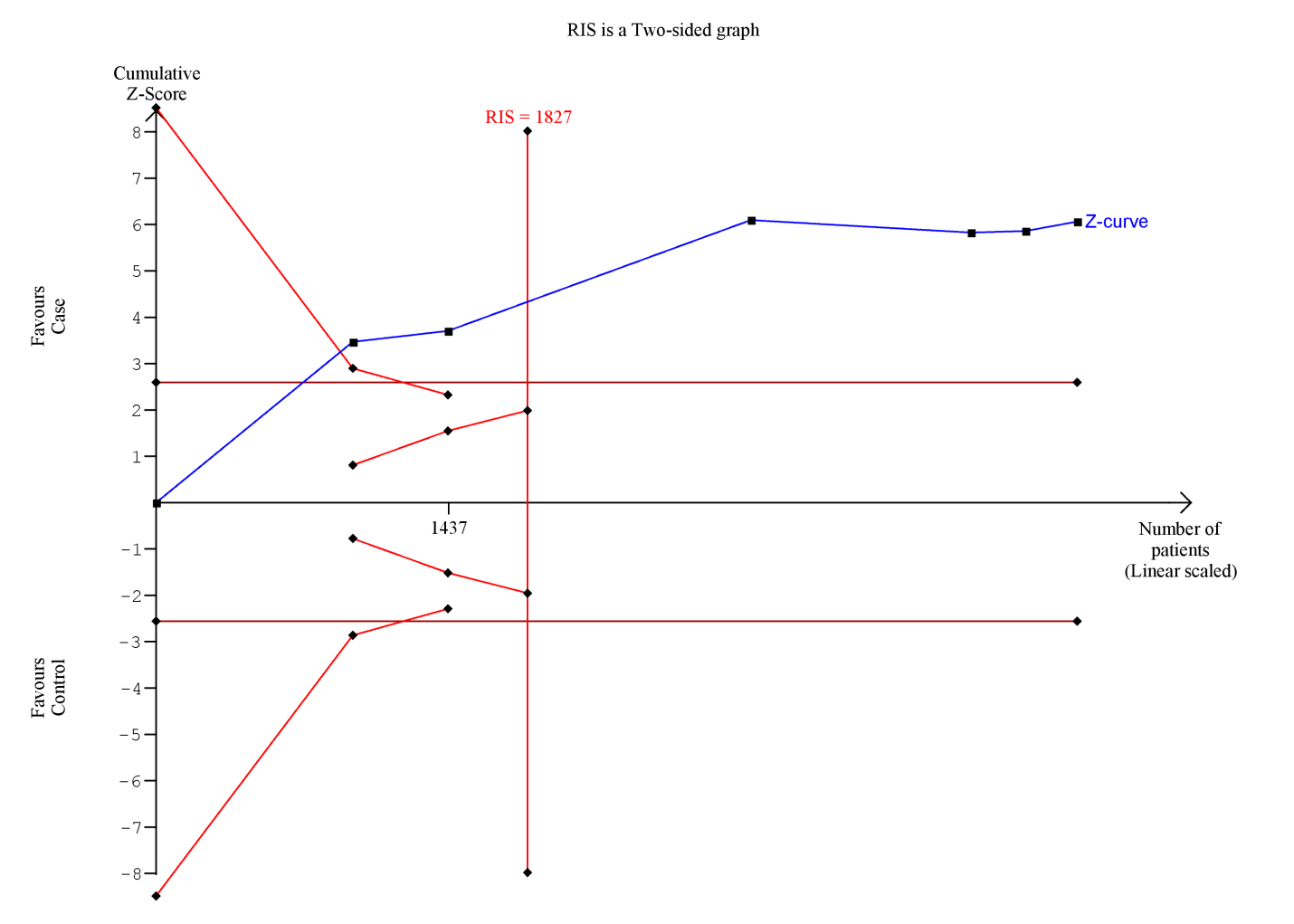
**

**Supplemental Digital Content 2. Figure S8**: Trial sequential analysis of association of *rs157580* polymorphism and the risk of Alzheimer's disease in heterozygous model.

**
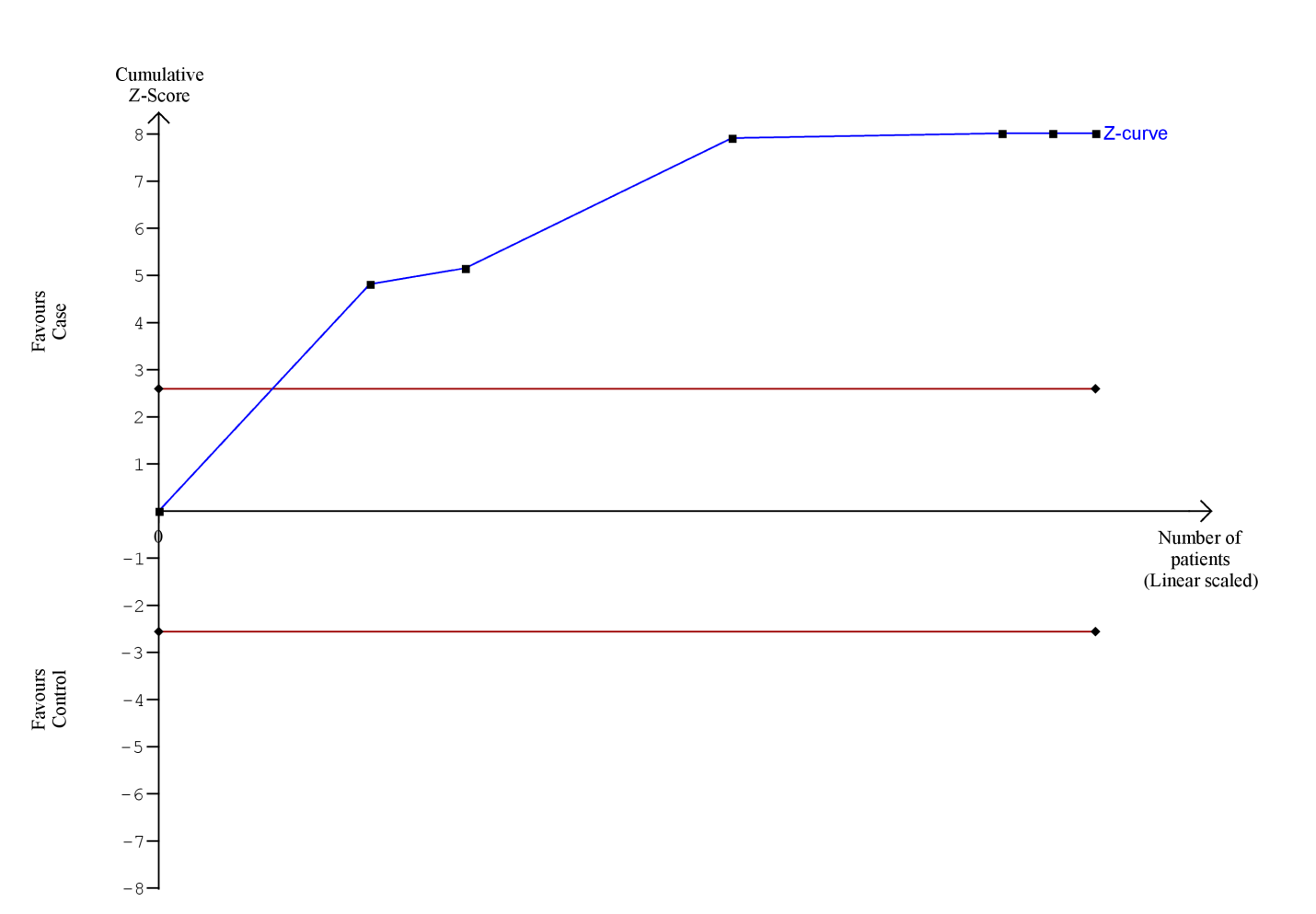
**

**Supplemental Digital Content 2. Figure S9**: Trial sequential analysis of association of *rs157580* polymorphism and the risk of Alzheimer's disease in dominant model.

**
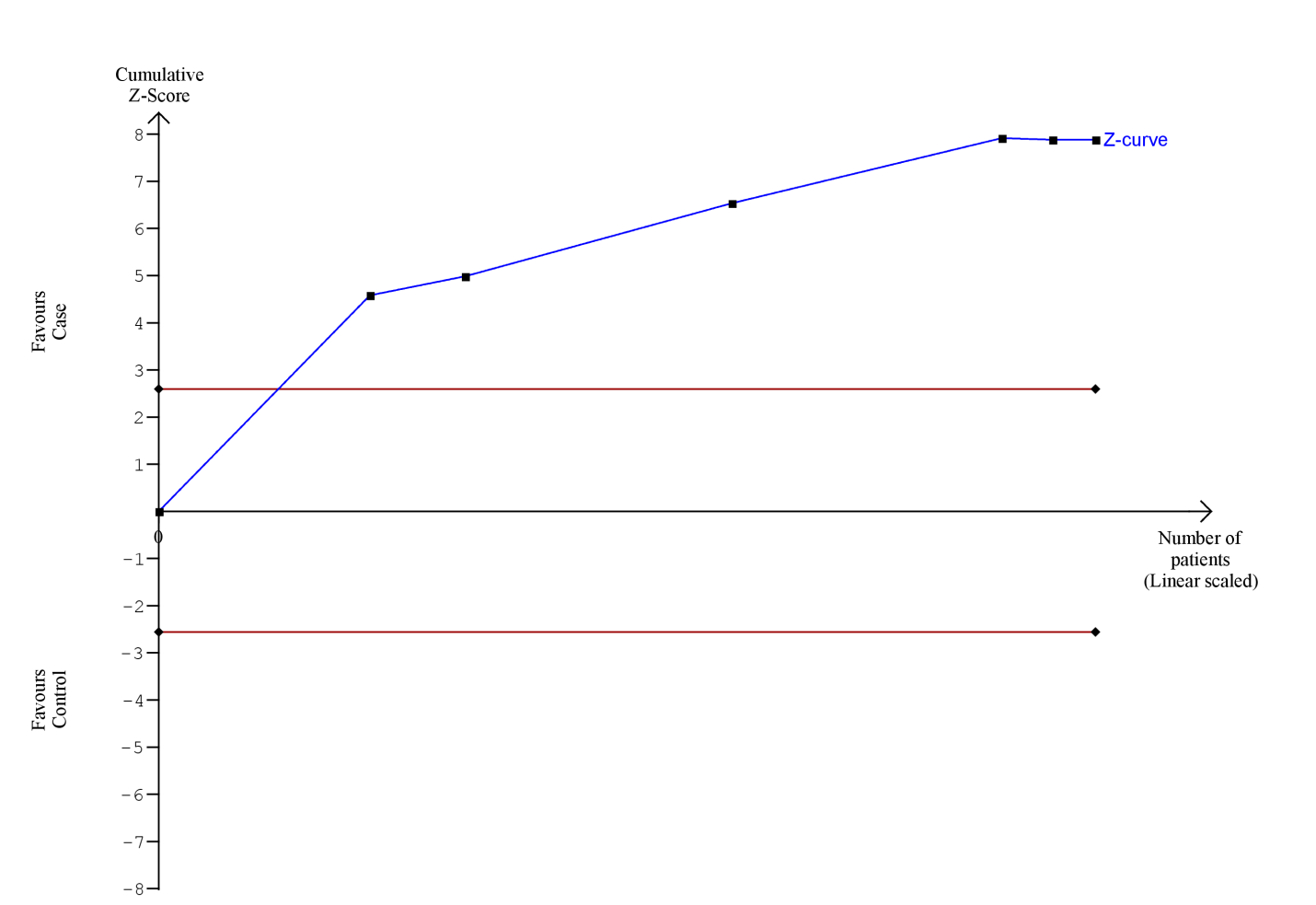
**

**Supplemental Digital Content 2. Figure S10**: Trial sequential analysis of association of *rs157580* polymorphism and the risk of Alzheimer's disease in recessive model.
